# Eye metrics often reflect visual conscious awareness, conscious content, and neural processing in cerebral blindness

**DOI:** 10.1038/s42003-025-08945-5

**Published:** 2025-12-01

**Authors:** Sharif I. Kronemer, Victoria E. Gobo, Shruti Japee, Elisha P. Merriam, Benjamin Osborne, Peter A. Bandettini, Tina T. Liu

**Affiliations:** 1 Laboratory of Brain and Cognition (LBC), National Institute of Mental Health (NIMH), National Institutes of Health (NIH), Bethesda, MD USA; 2 Department of Neurology and Ophthalmology, Medstar Georgetown University Hospital, Washington, DC USA; 3 Functional MRI Facility, NIMH, NIH, Bethesda, MD USA; 4 Department of Neurology, Georgetown University Medical Center, Washington, DC USA

**Keywords:** Perception, Consciousness, Stroke

## Abstract

Cerebral blindness is caused by damage to the primary visual pathway. Some people with cerebral blindness retain degraded vision and non-visual sensations and can perform visually guided behaviors within their blind visual field. These cases raise questions about visual conscious perception and residual neural processing in cerebral blindness. A major challenge in this research is that subjective reporting on experiences in the blind field can be unreliable. Alternatively, eye metrics offer a promising objective marker of conscious awareness, conscious content, and brain activity. In this study, we recorded visual stimulus-evoked pupil size, blink, and microsaccade responses in neurotypical participants and both the sighted and blind fields of cerebrally blind participants. For most patients, we found that eye metrics inferred conscious awareness in the blind field. Also, pupil size responded to both real and illusory stimulus luminance in the sighted field but not in the blind field. Furthermore, eye metrics were linked to visual stimulus-evoked occipital cortical field potentials in the blind field, suggesting residual cortical processing. These findings support eye metrics as an indicator of visual conscious perception and neural processing in cerebral blindness, with potential applications for tracking vision recovery following damage to the primary visual pathway.

## Abstract (web summary)

Investigating stimulus-evoked pupil size, blinking, and microsaccade dynamics as indicators of conscious awareness, conscious content, and visual sensory cortical processing in the blind visual field of people with cerebral blindness.

## Introduction

A person with cerebral blindness experiences partial or complete loss of conscious vision following a lesion to the visual pathways posterior to the lateral geniculate nuclei (e.g., optic radiations or primary visual cortex)^1,2^. A longstanding question in cerebral blindness is whether visual neural processing persists, particularly through secondary visual pathways such as the tectopulvinar pathway^3,4^, and whether this residual activity gives rise to visual conscious awareness or behavior. We define visual conscious awareness as conscious access or the subjective experience of visual stimulation *independent* of its specific phenomenal attributes or conscious content (i.e., “what it feels like”). This broader definition accounts for reports in cerebral blindness of vaguely visual and non-visual conscious experiences (i.e., the awareness of “something” without the phenomenal characteristics associated with normal conscious vision). Understanding the nature of residual awareness can illuminate how primary and secondary visual pathways contribute to both conscious and unconscious vision and behavior in cerebral blindness.

Motivating these questions are reports of cerebrally blind people who can respond to visual stimulation and perform visually guided behaviors in their blind field, such as detecting movement and changes in environmental luminance, orienting towards visual stimuli, or navigating obstacle courses^5–7^. Similar findings are also reported in non-human primates with ablated primary visual cortex^8–10^. For many cerebrally blind people, accurate blind field performance on visual tasks corresponds with visual conscious awareness, including degraded abnormal vision and non-visual “feelings” and “sensations”^11–13^. However, a subset of cerebrally blind people appears to exhibit *blindsight*: the preservation of visually guided behaviors *without* visual conscious awareness^14–17^. When instructed to perform a visual task, a person with blindsight will respond with confusion (e.g., “How can I look at something I haven’t seen?”), and are often surprised by their above chance performance because their actions felt random or by complete guessing^4,6^.

While blindsight is often cited as evidence that visual processing can occur in the absence of conscious awareness, it remains controversial whether people with blindsight genuinely experience complete unconscious vision without any blind field visual conscious awareness^18^. Partly fueling this debate are reports that residual conscious vision and degraded abnormal vision in cerebral blindness can be neglected by standard visual tasks and questionnaires (e.g., did you *see* or *not see* the image?)^13,19^. For instance, a person might indicate being unable to “see” anything, yet consciously aware of its presence and visual characteristics (e.g., location, size, and color). These experiences challenge narrow definitions of conscious awareness and underscore the limits of relying solely on subjective report for disclosing consciousness^20^. To address this limitation, an objective measure of conscious perception may assist in probing blind field visual conscious awareness independent of its phenomenal attributes. Such markers could also help infer conscious content, including distinguishing among normal conscious vision, degraded conscious vision, and non-visual conscious experiences.

A promising marker of visual conscious awareness and linked residual neural processing in cerebral blindness is eye metrics, including pupil size, blinking, and eye movements (e.g., saccades and microsaccades). In healthy physiology, these measures are linked with brain activity across species^21–26^. Correspondingly, eye metrics are valuable to infer the consequences of central neural processing, including those related to conscious awareness. For example, pupil size, blink, and microsaccade responses have been used to predict conscious perception of near-perceptual threshold stimuli^23^. Furthermore, pupil size is indicative of conscious content, including the perceived or imagined brightness of an image, independent of physical luminance^27–32^, while eye movements can reflect the direction of perceived motion^33^.

In cerebral blindness, research linking eye metrics, conscious awareness, conscious content, and neural processing is limited. Most existing studies focus on pupillary responses to ambient light and visual stimuli, such as grating images, which are often preserved in the blind field^11,34,35^. Likewise, pupillary responses to visual stimuli also predict conscious vision and blindsight in cerebral blindness^12,36^. Pupil size change is also responsive to complex visual features in healthy and cerebrally blind people. For instance, blind field pupillary responses (and facial muscle activity) were present for affective images of human facial expressions and body gestures^37^. Meanwhile, less is known about blinking and eye movements in cerebral blindness. Previous studies find the maintenance of the blind field blink reflex, optokinetic nystagmus, and stereopsis^6,38^, but there are conflicting results^35,38^.

In the current experiment, we examined whether visual stimulus-evoked pupil size, blinking, and microsaccades can serve as objective markers of conscious awareness, conscious content, and residual neural processing in cerebral blindness. Patient participants—people with cerebral blindness—viewed images with real (i.e., physically present) and illusory brightness (the glare illusion)^39,40^ designed to disentangle eye metrics linked with conscious versus unconscious neural processing. Alongside patients, we also tested age and education-matched control participants as a healthy vision comparison group. Finally, we recorded cortical activity with magnetencephalography (MEG) to relate behavior and eye metrics with cortical processing of visual stimuli.

We hypothesized that the control and patient participants would exhibit similar visual stimulus-evoked pupil size, blink, and microsaccade responses for stimuli presented in the sighted field. In the blind field, we predicted that eye metrics would reflect residual visual conscious awareness, including the experience of residual and degraded conscious vision and non-visual sensations. Furthermore, we expected patients with residual conscious awareness to show a pupillary light response to both real and illusory bright stimuli, while those without blind field conscious awareness would only show a pupillary response to physical brightness. These anticipated outcomes are important for establishing eye metrics as indicators of both conscious and unconscious visual processing in cerebral blindness.

## Results

### Visual behavior

**Fig. 1:**
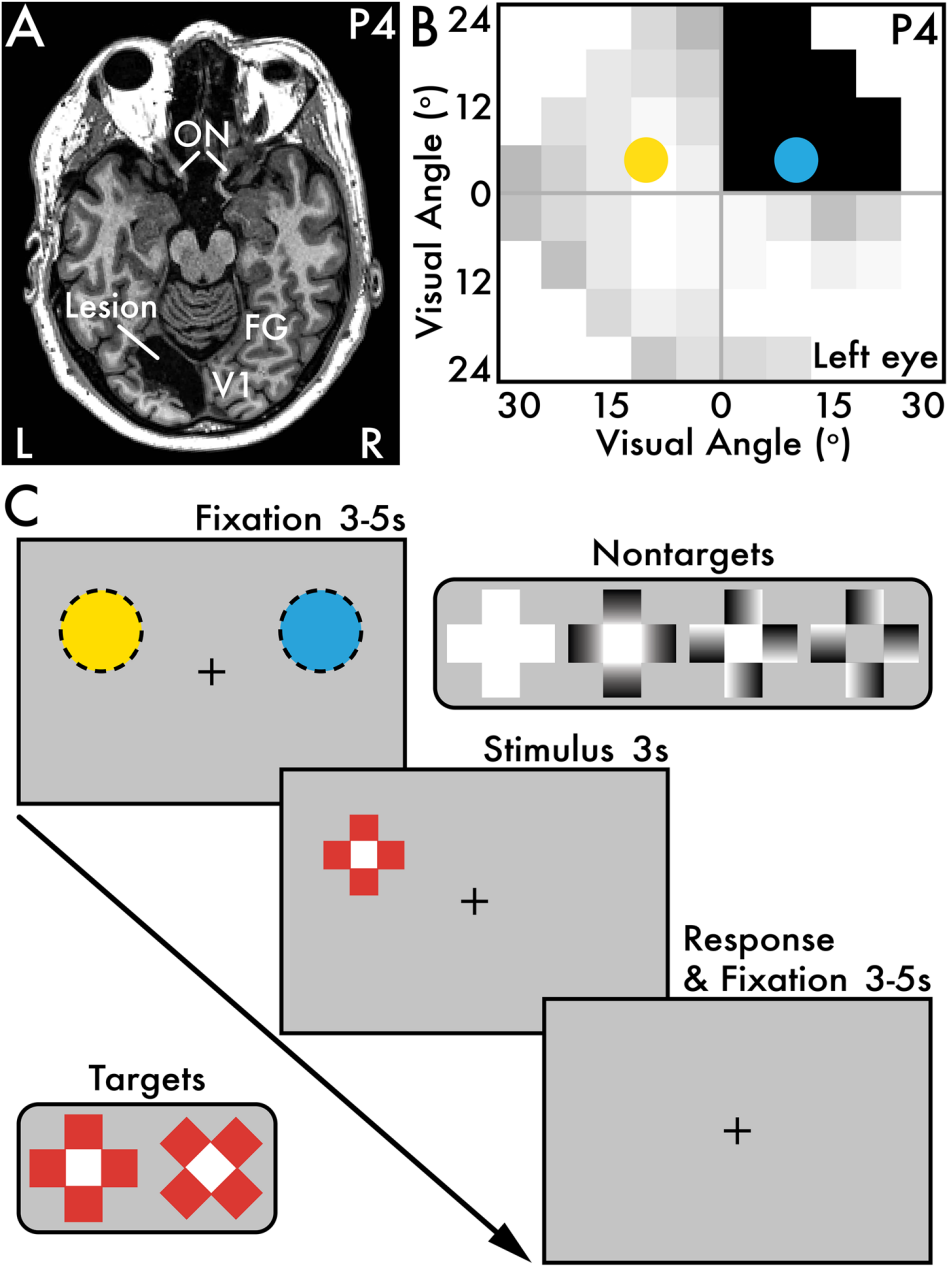
*Patient participant P4 stroke location and Humphrey visual field test results, and the visual perception task.* **A** Patient participant P4 (Table 1) axial T1-weighted whole brain anatomical MRI scan revealing a lesion in the left primary visual cortex (V1) affecting regions ventral to the calcarine sulcus. The right (R) cortical hemisphere is intact. Major primary visual pathway anatomical landmarks are labeled: optic nerve (ON), V1, and fusiform gyrus (FG). **B** P4’s Humphrey visual field (HVF) test results from the left eye in degrees (^o^) of visual angle indicates a right homonymous superior quadrantanopia. Sighted areas are indicated by white and light gray squares, while blind areas are indicated by dark gray and black squares. The colored circles (sighted field: yellow; blind field: blue) indicate the on-screen stimulus presentation locations for the visual perception task. **C** Each visual perception task trial involved three main phases: (1) a pre-stimulus fixation period (3–5 seconds [s]), (2) a stimulus presentation period (3 s), and (3) a post-stimulus response and fixation period (3–5 s). Throughout all phases within a trial, participants were instructed to maintain fixation on the central plus sign image. There were two categories of stimuli: (1) *target* and (2) *nontarget*. The target stimuli were red with a white center and appeared in equal proportion between two orientations: (1) plus sign (+) and (2) x-oriented. Participants were instructed to make an immediate keypress upon perceiving the target stimulus regardless of its perceived vividness (i.e., clearly versus barely seen or sensed) and indicate which orientation the target appeared. There were four nontarget stimuli that appeared in equal proportion: (1) white, (2) glare, (3) nonglare, and (4) isoluminant stimuli. Participants were instructed that no keypress was required when the nontarget stimuli appeared. The yellow (sighted) and blue (blind) dotted circles approximate the stimulus presentation locations for P4, corresponding with the circles overlaid on the HVF test results in **B**. The stimulus presentation locations were adjusted for each patient participant according to their visual field impairment (Table 1; Supplementary Fig. 1). Control participants were shown stimuli in the exact same on-screen locations as their paired patient participants (Table 2). Stimuli appeared in equal proportion between the two stimulus presentation locations.

Patient and control participants (Tables 1 and 2) were administered a visual perception task (Fig. 1C). For patients, visual stimuli were presented in both their sighted and blind visual fields (Supplementary Fig. 1), while all stimulus presentation locations were fully visible for controls. Participants also completed a brightness perception task that assessed the perceived real and illusory brightness of the visual perception task stimuli (Supplementary Fig. 2A). Full details of the task designs are provided in the *Visual Perception Task* and *Brightness Perception Task* Methods sections.

**Table 1:** *Patient participants’ demographic information (age and sex), eye recorded, etiology, visual impairment, and duration since injury onset*

Patient	Age (years)	Sex	Eye recorded	Etiology	Visual impairment	Onset (months)
P1	62	Male	Left	IS	LHH	10
P2	37	Male	Right	IS	LHIQ	9
P3	47	Male	Left	IS	RHH	31
P4	63	Male	Right	IS	RHSQ	10
P5	72	Female	Left	IS	LHH	29
P6	23	Female	Left	HS	RHH	39
P7	80	Male	Left	IS	LHIQ	21
P8	18	Male	Left	TBI	LHIQ	17

The participant’s age and the duration since injury onset are reported relative to the date of the first study session. Etiology: Ischemic stroke (IS); hemorrhagic stroke (HS); traumatic brain injury (TBI). Visual impairment: Left homonymous hemianopia (LHH); right homonymous hemianopia (RHH); left homonymous inferior quadrantanopia (LHIQ); right homonymous superior quadrantanopia (RHSQ).

**Table 2:** *Control participants’ demographic information (age and sex), eye recorded, and their paired patient participants*

Control	Age (years)	Sex	Eye recorded	Paired patient
C1	63	Female	Right	P1
C2	29	Male	Right	P2
C3	50	Male	Right	P3
C4	62	Female	Left	P4
C5	59	Male	Left	P5
C6	23	Female	Left	P6
C7	64	Female	Left	P7
C8	22	Male	Left	P8

The participant’s age is reported relative to the date of the study session. See Table 1 for information on the paired patient participants.

#### Blind aware and unaware patient participants

All patient participants reported instances of blind field conscious vision or non-visual sensations related to stimulus presentation, though with varying frequency (Tables 3 and 4). Patient participants were categorized as either *blind aware* or *blind unaware* based on their verbal report and behavioral performance. The blind *aware* patient participants (P2, P4, P7, and P8) frequently (predetermined criterion: >10% of trials; see *Correspondence among blind field task behavior, verbal perceptual report, and eye metric responses* Methods section) reported conscious awareness in the blind field, including statements such as “barely could see images” (P2), “I could sense there was movement” (P4), “confident about color but not shape” (P7), and “barely only able to catch the left [blind field] images” (P8; Table 3). In contrast, the blind *unaware* patient participants (P1, P3, P5, and P6) infrequently (predetermined criterion: <10% of trials; see *Correspondence among blind field task behavior, verbal perceptual report, and eye metric responses* Methods section) reported conscious awareness in the blind field (Table 4). In some cases, blind field conscious awareness for blind unaware patient participants were related to eye movements (i.e., making saccades away from central fixation to directly foveate on a stimulus). To evaluate the influence of conscious awareness, stimulus-evoked eye metrics in the blind field were analyzed separately for the blind aware and unaware patient groups.

**Table 3:** *Summary of blind aware patient participants’ blind field task behavior, eye metrics, and verbal perceptual report results*

Blind aware				
Patient	Task behavior	Eye metrics	Verbal report	Verbal report summary
P2	Present	Present	Present	*Summary:* Reported seeing or sensing most task stimuli. *Representative quotes:* “I can see it [images] but not processing”; “I can feel something is there but cannot tell you where it is or what it is”; “the left [blind field] images were incomplete like a bad signal”; “takes time to perceive them and figure out the shape”; “barely could see images on the left [blind field]”; “images kind of obscured – not in focus – on the left [blind field] but could see something was there”.
P4	Present*	Present	Present	*Summary:* Reported seeing or sensing most task stimuli. *Representative quotes:* “I could sense there was movement in the upper right [blind field] but could not see anything”; “feeling of movement [or] clicking down in the upper right quadrant [blind field] … but no vision”; “[blind field looked like] static noise; old time TV”; “feels like an area with static noise would be added when there was not any before”; “[had sensation of images akin to movement but] cannot see what it is”; “[sensation of images were] like a vibration”; “strong feeling”.
P7	Present	Present	Present	*Summary:* Reported seeing most task stimuli. *Representative quotes:* “[images] more faded and lacked color [in the blind field]”; “[the images were] a little more washed out [in the blind field]”; “[images] just did not look as sharp on the left [blind field]”; “[images have] more contrast and brighter on the right [sighted field]”; “confident about color but not shape [of images in the blind field]”; “more aware of images on the right [sighted field]”.
P8	Absent	Present	Present	*Summary:* Reported seeing most task stimuli. *Representative quotes:* “felt there was a fog over the left side [blind field]”; “could not tell the shape [of images]”; “not sure if I mistook a gray for red [image]”; “barely only able to catch the left [blind field] images”; “left [blind field] images were smudged”; “could catch a few on the left [blind field] but cannot really tell”; “surprised that when I looked on the left [blind field] that there was a red image – thought it was black”; “on the left [blind field] what might be red is grayish”.

*Patient participant P4 (Table 1) task behavior was absent in the original visual perception task but present in the behavioral-adapted task (Fig. 6B versus C). See *Correspondence among blind field task behavior, verbal perceptual report, and eye metric responses* Methods section for details for how “present” and “absent” designations were determined.

**Table 4:** *Summary of blind unaware patient participants’ blind field task behavior, eye metrics, and verbal perceptual report results*

Blind unaware				
Patient	Task behavior	Eye metrics	Verbal report	Verbal report summary
P1	Absent	Absent	Absent	*Summary:*~10 instances of seeing or sensing task stimuli. *Representative quotes:* “might have felt [an image] but so faint and fast”; “saw a faint gray [image]”; “might have noticed a red or gray image [but might be] my mind playing tricks on me”; “might have seen silver images on the left [blind field] but just might have been my brain”.
P3	Absent	Absent	Absent	*Summary:* ~6 instances of seeing or sensing task stimuli. *Representative quotes:* “I did not see anything to my right [blind field] but had the feeling to look to the right as if something might have appeared”; “an image on the right [blind field] just appeared in my view”; “it [the image] kind of popped up”.
P5	Absent	Present	Absent	*Summary:* ~12 instances of seeing task stimuli. *Representative quotes:* saw “very slight shadows”, “white shadow”, “red tint”, and “red shadow on the left [blind field]”; “could see red [in the blind field] but not sure what it was”; “saw red [in the blind field] but could not tell the shape”.
P6	Absent	Absent	Absent	*Summary:*~9 instances of seeing or sensing task stimuli. *Representative quotes:* “felt once that there was something on the right [blind field]”; “felt an image [in the blind field] but not sure; was not vision”.

Note that verbal report was considered absent when visual conscious awareness was reported in <10% of trials. Some instances of blind field conscious awareness were linked to eye movements away from central fixation. See *Correspondence among blind field task behavior, verbal perceptual report, and eye metric responses* Methods section for details for how “present” and “absent” designations were determined.

#### Visual perception task

**Fig. 2:**
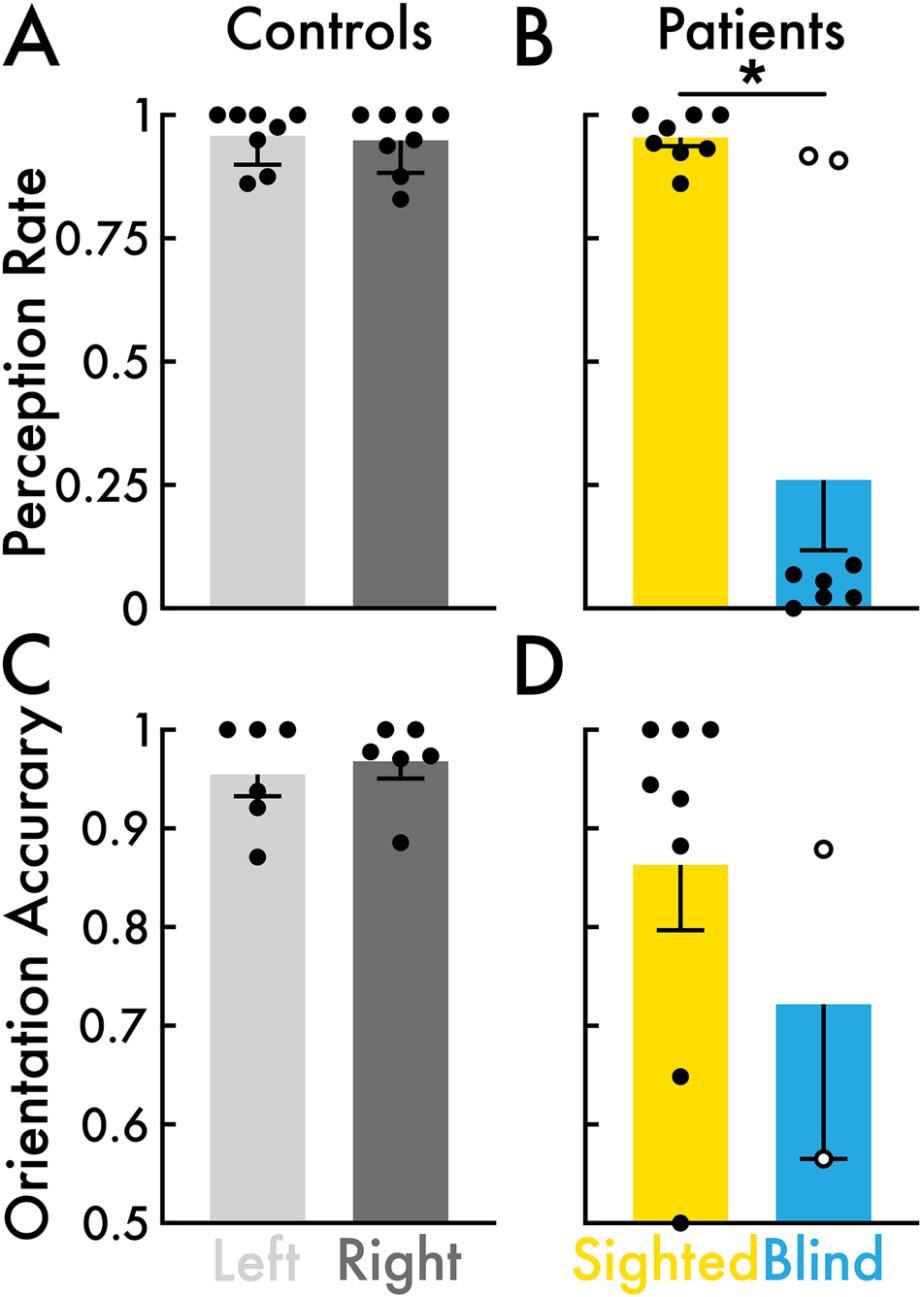
*Visual perception task behavioral results.* **A** Target stimulus perception rate for the left (light gray) and right (dark gray) visual fields in control participants (*N* = 8). Left versus right visual field perception rates were not significantly different. **B** Target stimulus perception rate for the sighted (yellow) and blind fields (blue) in patient participants (*N* = 8). Open circles highlight patient participants P2 and P7 who reported high (>0.9) target stimulus perception rate in their blind field. Sighted versus blind field perception rates were significantly different (*p < 0.05). **C** Target stimulus orientation discrimination accuracy for the left and right visual fields in control participants (*N* = 6). Accuracy could not be calculated for control participants C1 and C2 (Table 2) who completed an early version of the visual perception task (see *Visual Perception Task* Methods section) that did not include a target orientation discrimination component. Left versus right visual field accuracy were not significantly different. **D** Target stimulus orientation discrimination accuracy for the sighted (*N* = 8) and blind fields (*N* = 2) in patient participants. Blind field accuracy was only calculated for patient participants P2 and P7 with high blind field perception rate (highlighted with open circles in **B**). Sighted versus blind field accuracy was not statistically evaluated. In all subplots, the open and closed circles represent individual participants, and the bars and error bars indicate the group mean and standard error of the mean, respectively.

Participants were instructed to report the visual perception or sensation for the presentation of target stimuli (Fig. 1C). Control participants performed with high target stimulus perception rate (left visual field mean rate = 0.96; right visual field mean rate = 0.95; Fig. 2A) and high target stimulus orientation discrimination accuracy (left visual field mean rate = 0.95; right visual field mean rate = 0.97; Fig. 2C). There were no significant differences (*p* > 0.05) in either perception rate or orientation discrimination accuracy between the left versus right visual fields.

For stimuli presented in the sighted field, patient participants performed with high target stimulus perception rate (mean rate = 0.95; Fig. 2B) and high target stimulus orientation discrimination accuracy (mean rate = 0.86; Fig. 2D). However, for target stimuli presented in the blind field, the patient participants performed with low perception rate (mean rate = 0.26; Fig. 2B). Excluding P2 and P7, who both had blind field perception rates greater than 0.9, the group mean perception rate for target stimuli in the blind field dropped to near zero (0.043). The blind field perception rate was significantly less than the sighted field (*p* = 0.016). The blind field target stimulus orientation discrimination accuracy was 0.88 and 0.57 for P2 and P7, respectively (Fig. 2D).

#### Brightness perception task

Control and patient participants reported similar perceived brightness of the glare, nonglare, and isoluminant stimuli (Supplementary Fig. 2B, C). A statistically significant effect of stimulus type on perceived brightness was found for control (χ^2^(2) = 14.25, *p* < 0.0001) and patient participants (χ^2^(2) = 12.97, *p* = 0.0003). The glare stimulus was reported as significantly brighter than the nonglare stimulus for control (*p* = 0.016), but not patient participants (*p* > 0.05). The nonglare stimulus was reported as significantly brighter than the isoluminant stimulus for control (*p* = 0.008) and patient participants (*p* = 0.008).

### Eye metrics

#### Target stimuli

Control participants showed robust changes in pupil size, blink rate, and microsaccade rate for target stimuli compared to corresponding blank events. At the group-level, target stimuli elicited a unimodal pupil dilation after stimulus onset, blink rate suppression after stimulus onset and blink rate enhancement after stimulus offset, and microsaccade rate suppression both after stimulus onset and offset (Supplementary Fig. 3A).

Control participant group-level target stimulus-evoked eye metric responses yielded high, statistically significant epoch-level prediction accuracy for distinguishing target stimulus from blank event epochs based on pupil size, blink, and microsaccade responses for both the left (mean accuracy = 0.79; *p* = 0.004) and right visual field (mean accuracy = 0.83; *p* = 0.004; Supplementary Fig. 5A). The left versus right visual field accuracy did not statistically differ (*p* > 0.05), indicating that target stimulus-evoked eye metric responses were consistent across hemifields.

Patient participants revealed similar pupil size, blink rate, and microsaccade rate changes as control participants for target stimuli presented in the sighted field (Supplementary Fig. 3A versus B). However, differences emerged in blind field responses. For blind aware patient participants, the blind field eye metric responses were reduced in amplitude and shorter in duration yet shared a similar profile (e.g., pupil dilation and microsaccade rate suppression following stimulus onset; Supplementary Fig. 3C). For blind unaware patient participants, there were no changes in pupil size, blink rate, or microsaccade rate (Supplementary Fig. 3D).

Patient participant group-level target stimulus-evoked eye metric responses yielded high, statistically significant epoch-level prediction accuracy for distinguishing target stimulus from blank event epochs based on pupil size, blink, and microsaccade responses in the sighted field (mean accuracy = 0.83; *p* = 0.004; Supplementary Fig. 5B). Accuracy was not statistically different from chance in the blind field for blind aware and unaware patient participants, and blind field accuracy did not statistically differ between blind aware versus unaware patient participants (*p* > 0.05). The sighted field accuracy was statistically greater than in the blind field (*p* = 0.008; Supplementary Fig. 5B).

#### Nontarget stimuli

**Fig. 3:**
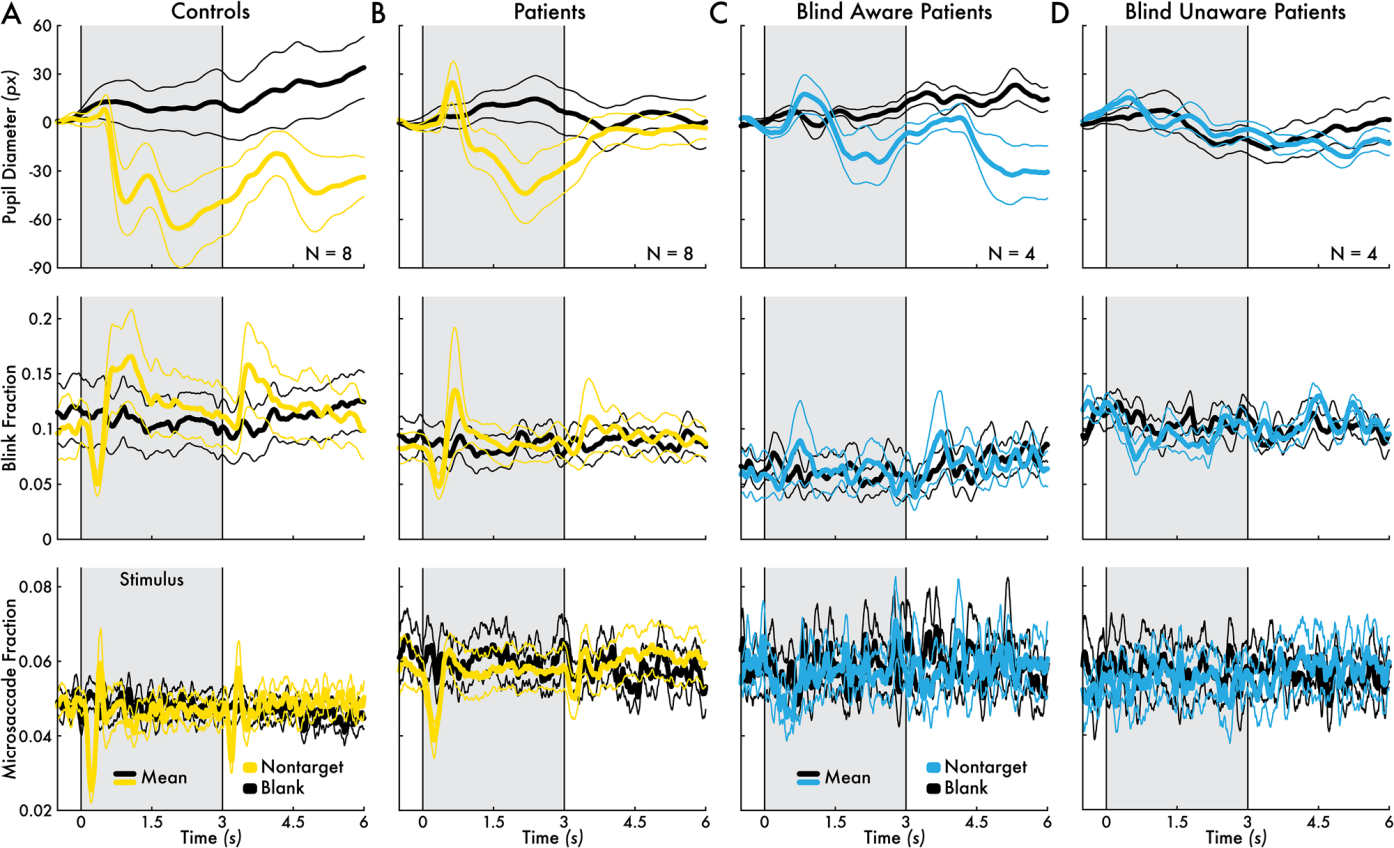
*Nontarget stimulus-evoked pupil, blink, and microsaccade responses.* Pupil diameter, blink fraction, and microsaccade fraction change preceding (0.5 seconds [s]) and following (6 s) the nontarget stimuli (averaged across the white, glare, nonglare, and isoluminant stimuli; see *Visual Perception Task* Methods section; yellow = sighted visual field; blue = blind visual field) or blank event (black) for **A** control participants (*N* = 8) averaged between the left and right visual fields, **B** patient participants (*N* = 8) sighted field, **C** blind aware patient participants (*N* = 4) blind field, and **D** blind unaware patient participants (*N* = 4) blind field. The group mean eye metric timecourses are shown (thicker traces) bounded by the standard error of the mean (thinner traces). The 3-s stimulus presentation interval is highlighted by a gray area bounded between two vertical lines at 0 and 3 s.

Control participants showed robust changes in pupil size, blink rate, and microsaccade rate for nontarget stimuli (average response across glare, nonglare, isoluminant, and white stimuli) versus corresponding blank events (Fig. 3A). Specifically, nontarget stimuli induced sustained pupil constriction after stimulus onset, blink rate suppression and enhancement after stimulus onset and blink rate enhancement after stimulus offset, and microsaccade rate suppression and enhancement after stimulus onset.

**Fig. 4:**
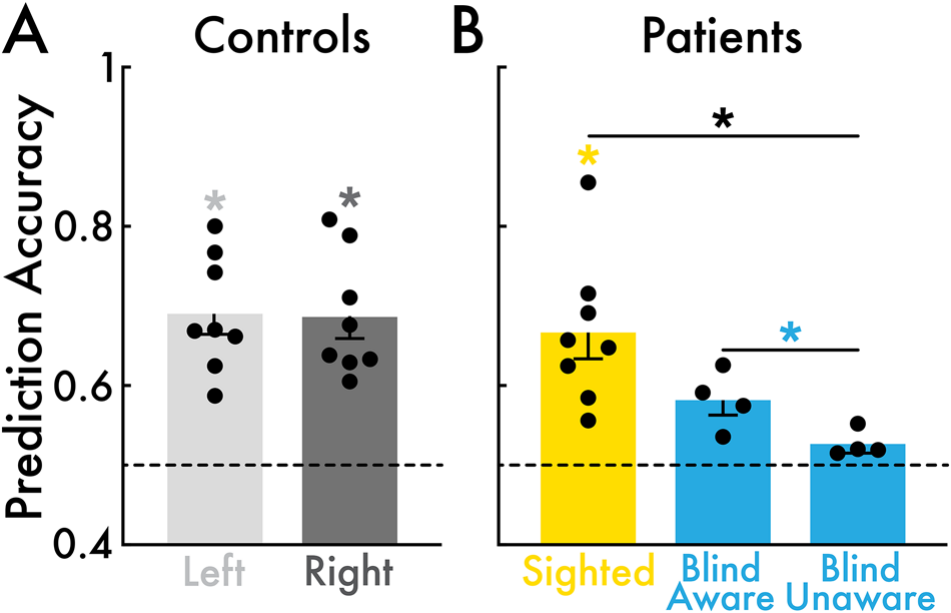
*Eye metric-based nontarget stimulus versus blank event classification performance.* **A** Prediction accuracy for the left (light gray) and right (dark gray) visual fields in control participants (*N* = 8). Accuracy in the left and right visual fields was significantly greater than chance (*p < 0.05). Left versus right visual field accuracy was not significantly different. **B** Prediction accuracy for the sighted (yellow) and blind fields (blue) in patient participants (*N* = 8; blind aware patient participants: *N* = 4; blind unaware patient participants: *N* = 4). Accuracy was significantly greater than chance for the sighted field but not significant for the blind field for the blind aware and unaware participants. Sighted field accuracy was significantly greater than the blind field. Blind field accuracy was significantly greater for blind aware than unaware participants. In all subplots, chance level was approximately 0.5 (highlighted with a dotted line), the circles represent individual participants, and the bars and error bars indicate the group mean and standard error of the mean, respectively.

Control participant group-level nontarget stimulus-evoked eye metric responses yielded moderate, statistically significant epoch-level prediction accuracy for distinguishing nontarget stimulus from blank event epochs based on pupil size, blink, and microsaccade responses for both the left (mean accuracy = 0.69; *p* = 0.004) and right visual field (mean accuracy = 0.69; *p* = 0.004; Fig. 4A). The left versus right visual field accuracy did not statistically differ (*p* > 0.05), indicating that nontarget stimulus-evoked eye metric responses were consistent across hemifields.

Patient participants showed similar pupil size, blink rate, and microsaccade rate changes as control participants for nontarget stimuli presented in the sighted field (Fig. 3A versus B). Eye metric responses included pupil constriction after stimulus onset, blink rate suppression and enhancement after stimulus onset and blink rate enhancement after stimulus offset, and microsaccade rate transient suppression and enhancement after stimulus onset (Fig. 3B). However, differences were observed in eye metric responses to non-target stimuli in the blind field. For blind aware patient participants, the blind field eye metric responses were reduced in both amplitude and response duration compared to the sighted field, although sharing a similar change profile (e.g., pupil constriction, blink rate enhancement, and microsaccade rate suppression following stimulus onset; Fig. 3C).

Patient participant group-level nontarget stimulus-evoked eye metric responses yielded moderate, statistically significant epoch-level prediction accuracy for distinguishing nontarget stimulus from blank event epochs based on pupil size, blink, and microsaccade responses in the sighted field (mean accuracy = 0.67; *p* = 0.008; Fig. 4B). Accuracy was not statistically different from chance in the blind field for blind aware and unaware patient participants (*p* > 0.05). However, blind field accuracy was significantly greater in the blind field for blind aware versus unaware patient participants (*p* = 0.029; Fig. 4B), suggesting that nontarget stimulus-evoked eye metric responses were specifically present for blind aware participants. The sighted field accuracy was statistically greater than in the blind field (*p* = 0.004; Fig. 4B).

**Fig. 5:**
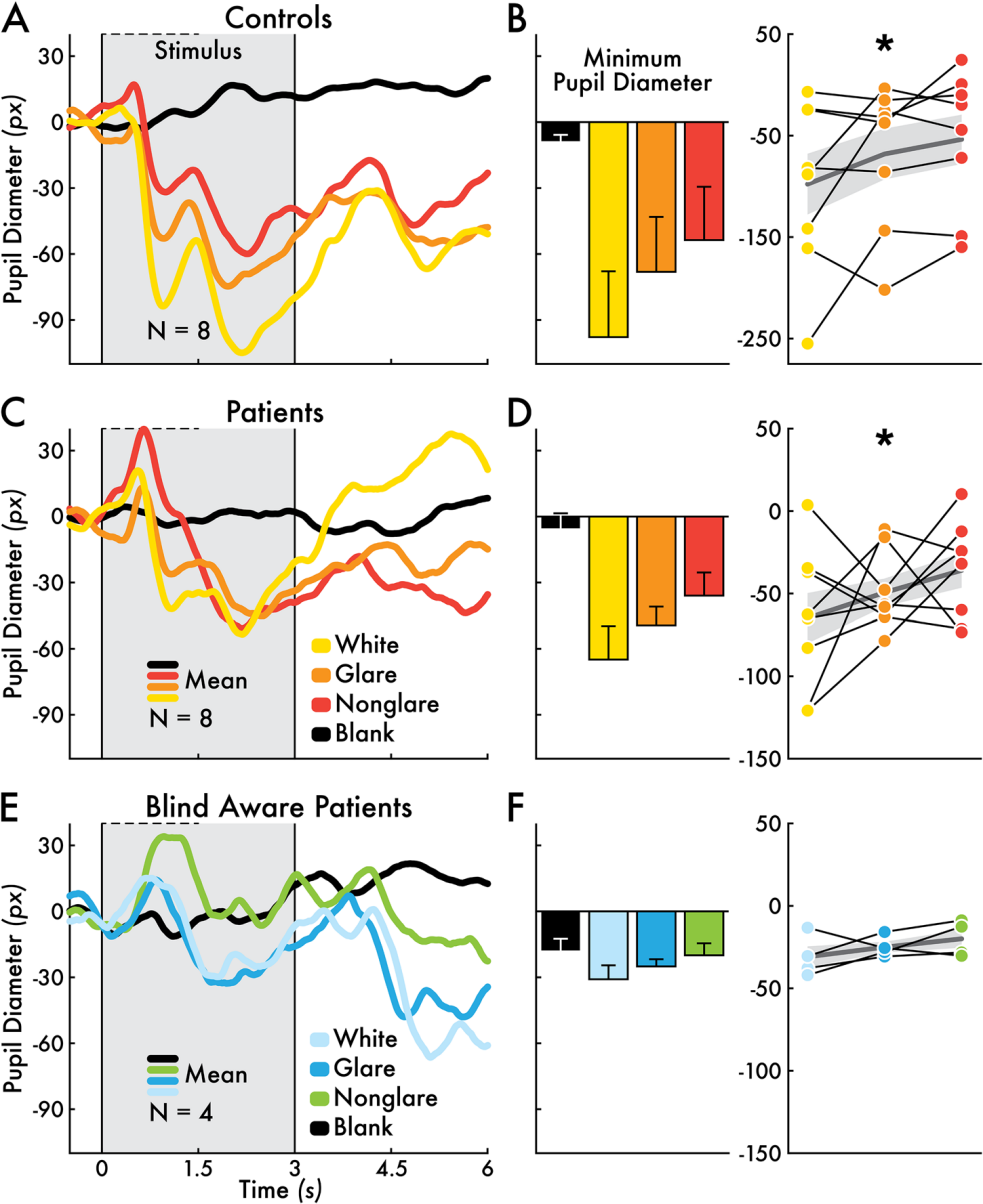
*White, glare, and nonglare stimulus-evoked pupil responses.* Pupil diameter change preceding (0.5 seconds [s]) and following (6 s) the white stimulus (yellow = sighted visual field; light blue = blind visual field), glare stimulus (orange = sighted visual field; blue = blind visual field), nonglare stimulus (red = sighted visual field; green = blind visual field), and blank event (black) for **A** control participants (*N* = 8) averaged between the left and right visual field, **C** patient participants (*N* = 8) sighted field, and **E** blind aware patient participants (*N* = 4) blind field. Blind unaware patient participants are not shown because there were no group-level stimulus-evoked pupillary responses. The eye metric timecourses represent the group mean response. The 3-s stimulus presentation interval is highlighted by a gray area bounded between two vertical lines at 0 and 3 s. The individual and group mean minimum pupil diameter change in the first 1.5 s post-stimulus (horizontal dotted line in **A**, **C**, and **E**) for **B** control participants (*N* = 8) averaged between the left and right visual fields, **D** patient participants (*N* = 8) sighted field, and **F** blind aware patient participants (*N* = 4) blind field. The bar heights indicate the group mean pupil diameter minimum with error bars showing the standard error of the mean (SEM). In the line plots, each circle corresponds with a participant and the black lines connect within participant pupil diameter minimum values across the white, glare, and nonglare stimuli. The group mean minimum pupil diameter and SEM are represented with a gray line and shaded gray background, respectively. The asterisks (*) indicate a statistically significant (*p* < 0.05) positive trend in minimum pupil diameter across stimulus types (i.e., greater pupillary constriction for the white versus both the glare and nonglare stimuli, and greater constriction for the glare stimulus versus the nonglare stimulus).

Finally, in both control participants and the sighted field of patient participants, pupillary constriction was modulated by real and illusory luminance. Significant pupil size differences were observed across the white, glare, and nonglare stimuli (controls: *p* < 0.012; patients: *p* < 0.027; Fig. 5A–D). The largest pupil constriction was elicited by the white stimulus and the smallest for the nonglare stimulus. In contrast, in the blind field of blind aware patient participants, pupillary constriction did not differ across the white, glare, and nonglare stimuli (*p* > 0.05; Fig. 5E and F). This lack of differentiation may reflect the atypical blind field conscious content, which patient participants described as degraded and non-visual. As a result, blind aware patient participants may have been unable to process the luminance properties of the stimuli (Table 3).

### P4 case study

Patient participant P4 illustrates the challenges of relying on self-report to assess visual conscious perception in cerebral blindness. At the same time, P4 highlights the utility of implementing eye metrics and possible other covert measures for detecting blind visual field conscious awareness. Moreover, P4 demonstrates how eye metrics may serve as indicators of residual neural processing of visual stimuli.

#### Visual behavior and eye metrics

**Fig. 6:**
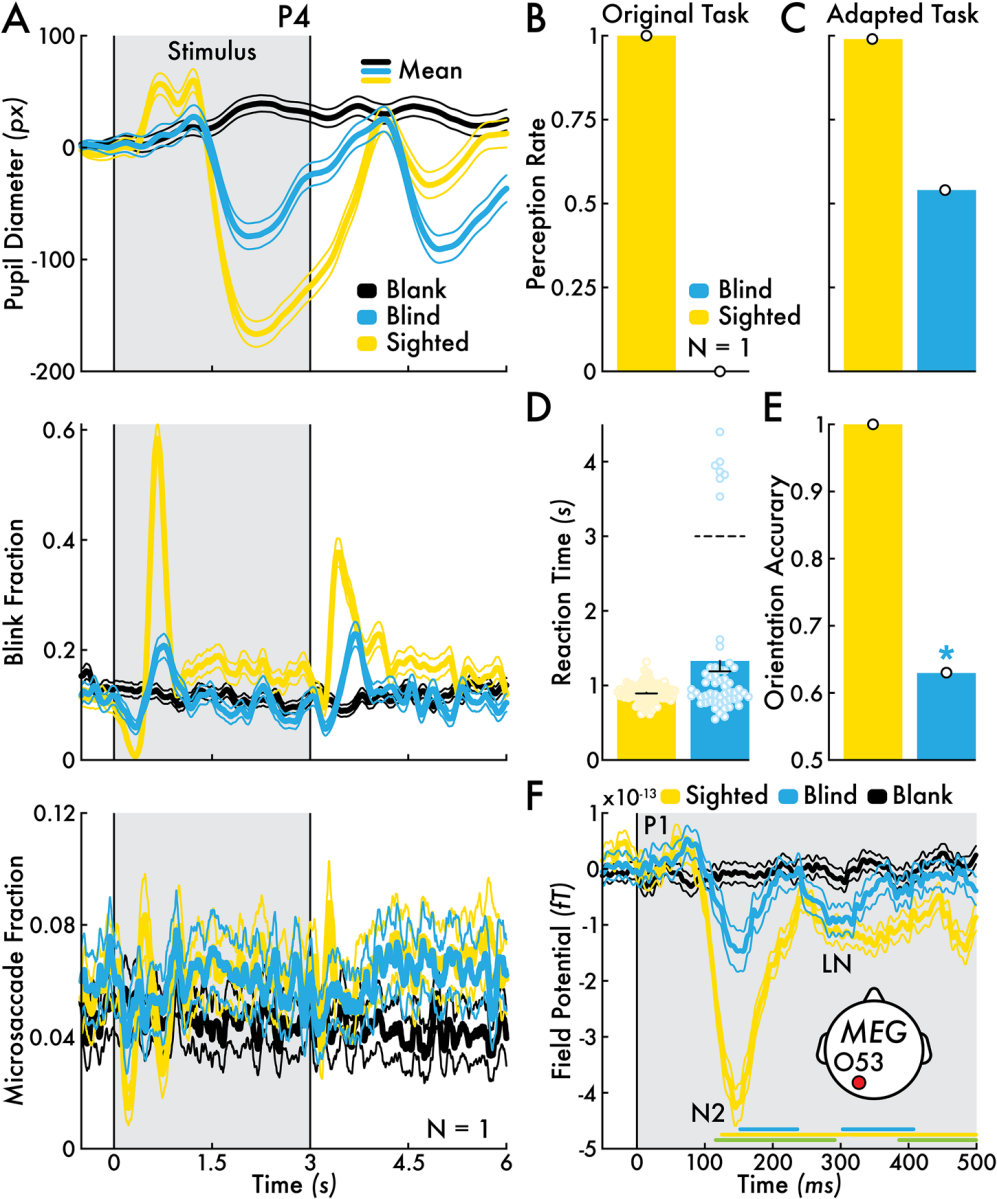
*Patient participant P4 sighted and blind field behavior, eye metric responses, and MEG responses for nontarget stimulus.* **A** Sighted (yellow) and blind visual fields (blue) pupil diameter, blink fraction, and microsaccade fraction change for nontarget stimulus versus blank event (black). The mean eye metric changes across all nontarget stimulus trials are shown (thicker traces) bounded by the standard error of the mean (SEM; thinner traces). **B** Perception rate for the target stimulus in the sighted (yellow) and blind fields (blue) for the *original* visual perception task (same result for P4 as depicted in Fig. 2B). **C** Perception rate for target stimulus in the sighted and blind fields for the *behavioral-adapted* visual perception task (see *Visual Perception Task* Methods section). **D** Target stimulus response reaction time in the sighted (*n* = 99 responses) and blind fields (*n* = 54 responses) for the behavioral-adapted visual perception task. The bar represents the mean reaction time with SEM. Sighted and blind reaction times were not significantly different. Individual trial reaction times are shown with open circles. A subset of responses (*n* = 7) may have corresponded with stimulus offset (horizontal dotted line). **E** Orientation discrimination accuracy for target stimuli in the sighted and blind field for the behavioral-adapted visual perception task. The blind field accuracy was significantly (*p < 0.05) above chance (0.5). **F** Nontarget stimulus and blank event evoked magnetencephalography (MEG) field potentials (femtotesla; fT) in the sighted (yellow) and blind fields (blue) for representative left occipital sensor O53 (see inset diagram for approximate sensor location on the scalp; see Supplementary Fig. 7 for results from all left and right occipital sensors). Major field potential components are highlighted: P1 (positive response near 100 milliseconds [ms]), N2 (negative response between 100 and 225 ms), and late negativity (LN; negative response that extends beyond 250 ms). Statistically significant intervals determined by cluster-based permutation testing are shown with horizontal lines for three evaluated contrasts: (1) sighted nontarget stimulus versus sighted field blank events (yellow), (2) blind nontarget stimulus versus blind field blank events (blue), and (3) sighted versus blind field nontarget stimulus (green). The mean field potential changes across all nontarget stimulus epochs are shown (thicker traces) bounded by SEM (thinner traces). The MEG results were acquired with the *MEG-adapted* visual perception task (see *Visual Perception Task* Methods section). Note that no error bars are depicted in subplots **B**, **C**, and **D** because the bars represent a single participant value: P4’s mean perception rate and accuracy.

P4 performed with a perception rate of 1 and 0 for target stimuli in the sighted and blind field, respectively, thus suggesting total blindness in his blind field (Figs. 2B and 6B). However, the presence of blind field, stimulus-evoked eye metric responses (see *Eye metrics* section; Fig. 6A) prompted a subsequent interview with P4 on his blind field visual conscious experiences. P4 described that he occasionally saw or felt “movement”, “clicking down”, “vibration”, or the addition of static noise in his blind field (Table 3). These experiences were distinct from normal conscious vision. As P4 explained, “I could sense there was movement in the upper right [blind field] but could not see anything”. Notably, P4 believed his blind field conscious experiences occurred randomly and did not correspond with visual sensory stimulation, including the stimuli presented in the visual perception task.

An adapted visual perception task was developed to probe whether P4’s blind field experiences corresponded with stimulus presentation (see *Visual Perception Task* Methods section). In the adapted task, P4 was instructed to press a key whenever he saw a target stimulus or experienced a sensation in his blind field (e.g., the feeling of movement), regardless to whether he believed those sensations were linked to task stimuli. This adapted instruction resulted in an increase of P4’s blind field perception rate from 0 in the original visual perception task to 0.54 in the adapted task (Fig. 6B versus C). P4’s blind field false positive rate (i.e., reporting a stimulus when none was present) was 0. Moreover, P4’s sighted versus blind field reaction time (RT) relative to stimulus onset were not statistically different (sighted field mean RT = 0.91 s; blind field mean RT = 1.33 s; *p* > 0.05; Fig. 6D). There was a subset of trials (*n* = 7) with prolonged RT, clustered at ~4 s post stimulus onset, possibly reflecting responses aligned with stimulus *offset*. These results indicated that P4’s blind field conscious experiences were linked to the presentation of task stimuli.

P4’s target stimulus orientation discrimination accuracy was 0.63 (34 correct responses out of 54 responses) and significantly above chance (*p* < 0.038; Fig. 6E). P4 indicated that he guessed the stimulus orientation by “instinct”, “inspired guess”, and by “an electric charge coming down my finger” so that even “before I could think, I pressed the button”. P4’s descriptions align with reports from other people with cerebral blindness who also achieve above chance performance on visually guided tasks by “only guessing”^4^.

When these behavioral findings were shared with P4, he was surprised and explained that he was previously unaware that he could “see” in his blind field. This case highlights that knowledge for the relationship between blind field conscious awareness, whether degraded vision or non-visual sensations, and visual events in the world is important for the recognition of residual visual conscious perception. Without this insight, people with cerebral blindness may ignore these experiences in daily life and fail to report them in the context of a clinical assessment or experimental task, just as P4 performed in the original perception task (Fig. 6B).

#### MEG

To assess whether P4’s blind field behavioral performance and stimulus-evoked eye metric responses corresponded with residual cortical processing, visual stimulus-evoked MEG field potentials were recorded. Scalp field potential changes over the left occipital cortex for nontarget stimuli presented in the sighted field included a small increase at 100 ms (first positivity or P1), a large negative deflection between 100 and 225 ms (N2), and a late negative (LN) shift after 250 ms (Fig. 6F; Supplementary Fig. 7A). Smaller in amplitude but similarly timed field potential changes were also found for the nontarget stimuli presented in the blind field. Scalp sensors over the right occipital cortex revealed weaker, but similarly timed responses, predominantly for stimuli presented in the sighted field (Supplementary Fig. 7B).

Cluster-based permutation testing on representative left occipital sensor O53 (likely responsive to neural activity in the left visual cortex; see Fig. 6F for approximate sensor location on the scalp) highlighted that the stimulus-evoked changes were greater than blank events for both the sighted and blind fields. However, the field potential response amplitude was significantly greater in the sighted versus blind field, particularly during the N2 and LN intervals. Interestingly, the sighted and blind field potential profile mirrors the sighted versus blind field eye metrics responses (Fig. 6A versus F). Two compatible explanations for the amplitude difference in the sighted and blind eye metric and MEG responses are: (1) the averaged physiological responses combine perceived and not perceived stimuli (i.e., P4 reported sensing ~50% of the stimuli presented in his blind field; Fig. 6C), and (2) impaired visual neural processing. Future studies that directly compare neural responses to perceived versus not perceived stimuli in the blind field of cerebrally blind people are necessary to resolve these alternative explanations, though not mutually exclusive (see *Future directions* Discussion section).

### Correspondence among blind field task behavior, verbal perceptual report, and eye metric responses

Task behavior, verbal report, and eye metrics in the blind field agreed in 6 patient participants: all measures were present (blind aware: P2, P4, and P7; Table 3) and all measures were absent (blind unaware: P1, P3, and P6; Table 4). There was disagreement among the measures for blind aware P8 and blind unaware P5.

For P8, both a pupillary response and verbal report were present (Supplementary Fig. 6A; Table 3), but task behavior indicative of blind field conscious awareness was absent (blind field perception rate = 0.06). However, P8’s verbal reports indicated that his poor behavioral performance was related to impaired color vision in his blind field (e.g., “what might be red is grayish”) making it difficult to discriminate between the red target and achromatic non-target stimuli (Fig. 1C; Table 3).

In contrast, P5 exhibited blink and microsaccade rate suppression following stimulus onset in the blind field that closely matched the amplitude and timing of the sighted field blink and microsaccade responses (Supplementary Fig. 6B). Nevertheless, P5 is categorized as blind unaware because she rarely reported visual conscious awareness in her blind field, including no instances of non-visual sensations of the task stimuli (e.g., “very slight shadows”; Table 4). If this categorization is accurate, P5’s blind field eye metric responses may be linked to unconscious, residual neural processing, suggesting that they do not always track conscious processing (see *Limitations* Discussion section).

## Discussion

We evaluated visual stimulus-evoked pupil size, blink, and microsaccade responses in healthy and cerebrally blind participants. A key finding was that eye metric responses were often but not always linked with visual conscious awareness in both the sighted and blind field. Specifically, eye metrics changes were present for stimuli presented in the blind field of blind aware patient participants. In contrast, at the group level, blind unaware patient participants showed no eye metric responses in the blind field. We also observed individual differences in stimulus-evoked eye metrics in the blind field. For instance, patient participant P8 retained pupil responses only (Supplementary Fig. 6A); P5 retained blink and microsaccade responses (Supplementary Fig. 6B); and P4 retained pupil, blink, and microsaccade responses (Fig. 6A). This result emphasizes the value of recording *multiple* eye metrics in the same person due to heterogenous responsiveness. A methodological contribution of this study is the implementation of machine learning-based classification methods to evaluate the presence of stimulus evoked eye metrics on an epoch-by-epoch basis (Supplementary Fig. 4). This approach is flexible, allowing for the assessment of multiple eye metrics and the inclusion of other covert measures of conscious awareness, and can be tailored to individual responsiveness.

Furthermore, the presence of stimulus-evoked eye metric responses in the blind field suggests residual neural processing of visual stimuli. Specifically, we found similar MEG field potential changes for visual stimuli presented in the sighted and blind fields, including the field components P1, N2, and LN (Fig. 6F; Supplementary Fig. 7A)^41^. The posterior scalp negativity (~250 ms post stimulus presentation) has been linked with visual conscious perception, while earlier and later brain potentials may be related to unconscious and post-perceptual processes^23,42^. These findings motivate future investigations on residual neural processing within the blind field and its relationship to conscious awareness and content (see *Future directions* section).

### Blind field conscious awareness and content in cerebral blindness

A longstanding controversy in cerebral blindness research is what exactly do these people experience in their blind field. Interrogating blind field conscious perception is challenging because residual and degraded conscious vision or non-visual sensations may be reported as no visual conscious awareness. Therefore, the method for inquiring on perceptual experiences in cerebral blindness is critical^19,43^. For instance, graded perceptual rating scales may be more sensitive than binary questionnaires (e.g., did you *see* something or not)^13,44^.

Our results highlight this concern. Patient participant P4 reported no visual conscious awareness in the original perception task. However, robust eye metric responses to stimuli presented in his blind field (Fig. 6A) prompted a subsequent interview and testing with an adapted perception task, both revealing consistent blind field conscious awareness for task stimuli that he described as a “feeling of movement” or “vibration” (Fig. 6B versus C; Table 3). P4 also performed above chance on blind field stimulus orientation discrimination, although he described his responses as guided by “instinct” or “inspired guess” (Fig. 6E). Notably, P4 was unaware these blind field conscious experiences were indicative of visual sensory stimulation.

This case illustrates a little discussed challenge for the methods of subjective report for measuring conscious awareness in people with cerebral blindness: individuals may be unaware that their blind field conscious experiences are related to visual stimulation, thereby they may ignore or be reticent to report on these instances of blind field conscious awareness. Correspondingly, these patients may indicate that they are blind because their blind field conscious experiences are not recognized as linked to visual stimulation. For patient participant P4, it was only when instructed to report *any* sensation in his blind field, regardless to whether he felt they were related to task stimuli, that a consistent association between his blind field conscious experiences and stimulus presentation emerged. Notably, this link had previously been unknown to P4 himself.

Similarly, patient participant P8’s performance on the visual perception task indicated little to no conscious vision in the blind field. However, P8 revealed by verbal report that he frequently perceived task stimuli in his blind field but was unable to distinguish the red target versus achromatic nontarget stimuli, so he decided not to respond to any stimulus during the task (Table 3). Even without P8’s verbal report, blind field conscious awareness could be inferred by a robust stimulus-evoked pupillary response that was similar to the sighted field pupil responses (Supplementary Fig. 6A). P4 and P8 highlight that eye metrics can assist the detection of blind field conscious awareness.

In addition, our results demonstrate that eye metrics can be used to infer conscious content in cerebral blindness. Specifically, we examined how pupil size is linked to real and illusory luminance or brightness. In the sighted field, we replicated previous findings that shows larger pupillary constrictions for stimuli with real and illusory brightness (the glare illusion; Fig. 5A–D)^29,45^; However, pupil size did not discriminate stimulus brightness—real or illusory—in the blind field of blind aware patient participants (Fig. 5E, F). This result is consistent with the verbal reports by blind aware patient participants who described their blind field conscious experiences as vague and devoid of visual detail (Table 3). Therefore, in the context of luminance, pupillary changes may provide insight into the visual conscious experience (e.g., its vividness) in response to visual stimulation in the blind field. Similarly, in a different context, pupillary responses have been shown to reflect the vividness of visual imagery^32^. In cases of cerebral blindness, degraded visual perception may limit the ability of eye metrics to discriminate real and illusory visual characteristics.

Finally, the absence of a pupillary light response to physical brightness in the blind field of blind unaware patient participants was unexpected. We hypothesized that a light-evoked pupil constriction would still be present in cerebral blindness and without conscious awareness. However, this result is consistent with prior findings that the pupillary light reflex may be impaired in cerebral blindness^35^. Moreover, the presence of this reflex may depend on the light stimulation parameters. For instance, a previous study found some people with cerebral blindness demonstrated a pupillary light response only after dark adaptation and to very bright light stimulation (e.g., direct sunlight)^34^. Therefore, the experimental stimuli in this study may have been insufficient to evoke a blind field pupillary light response without conscious awareness.

### Limitations

While our patient sample is diverse by age, education, and ethnicity, the current findings may not generalize to other people with cerebral blindness due to unique injury profile (e.g., lesion severity, duration, and location). Also, heterogenous responses across tasks and stimuli are a known challenge for establishing consensus in cerebral blindness research. In addition, stimulus features (e.g., size, duration, spatial frequency, luminance, color, and contour) can influence conscious awareness, conscious content, and eye metrics in cerebral blindness^4,12,34,46,47^. Thus, the current stimulus parameters may evoke unique behavior, eye metrics, and neural responses not replicable with other stimulus types or study designs.

A further limitation is that stimulus-evoked eye metric responses are not definitive of conscious awareness or content—they can be present and absent in both conscious and unconscious contexts^20,48,49^. For instance, some people with cerebral blindness show behavioral evidence of blindsight without corresponding pupillary responses^36^. Likewise, the participants in this study highlight the diverse relationships among eye metrics, behavior, and visual conscious perception. Stimulus-evoked eye metric responses were generally present for the blind aware and absent for the blind unaware patient participants. However, we observed that eye metrics were not in agreement with task behavior and verbal report in blind unaware patient participant P5: task behavior and verbal report indicated no blind field conscious awareness, while stimulus-evoked blink and microsaccade responses were present (Supplementary Fig. 6B; Table 4). This may be a case of stimulus-evoked eye metric responses linked to unconscious processing. However, we cannot rule out the possibility that the observed eye metrics in P5 indicate blind field conscious awareness, despite behavioral performance and verbal reporting that supported blindness.

In summary, eye metrics and other covert measures of consciousness should be considered as *one* source of evidence among other measures, including behavior and neuroimaging in the assessment of conscious awareness and content^20^. Likewise, the presence of neural activity patterns linked with conscious perception (e.g., N2; Fig. 6F) along with additional physiological markers that can be indicative of conscious awareness and content may help guide assessment of conscious perception in cerebral blindness.

### Future directions

The current investigation motivates future research, including studies with potential translational applications. First, it would be valuable to examine stimulus-evoked physiological responses, including eye metrics and neural activity associated with both consciously and unconsciously perceived stimuli *within* the blind field. For example, comparing neural activity associated with blind field perceived and not perceived visual stimulation may elucidate the neural mechanisms supporting visual conscious perception, including experiences described as non-visual sensations.

Second, recording eye metrics in cerebral blindness may be informative to track recovery or progress during vision rehabilitation. For example, the presence of stimulus-evoked eye metric responses and its activity profile (e.g., latency and magnitude) may infer the status of residual neural processing with or without conscious awareness. Moreover, changes in how eye metrics discriminate among visual features (e.g., stimulus luminance) may reflect a shift from non-visual or degraded conscious experience toward more typical visual perception.

## Conclusions

Probing visual conscious awareness, conscious content, and residual neural processing in cerebral blindness remains an active query. In the current study, pupil size, blink, and microsaccade responses are shown to be an accessible, objective measure of visual conscious awareness, conscious content, and brain processing in cerebral blindness. The results also highlight previous concerns that behavioral performance and self-reporting on a visual task may underestimate blind field conscious awareness, particularly those characterized as degraded abnormal vision and non-visual sensations. Importantly, each participant may exhibit a unique combination of eye metric responses, highlighting the value of recording multiple measures and using analyses that accommodate individual differences. Future translational applications of eye metrics in cerebral blindness includes tracking recovery of blind field visual neural processing with and without conscious awareness.

## Methods

### Participants

Eight cerebrally blind patient participants (females = 2; mean age = 50.25 years; standard deviation [SD] age = 22.76 years; mean education = 17.13 years; SD education = 2.59 years; Table 1) and eight age and education-matched (confirmed with Mann–Whitney U tests; *p* > 0.05) healthy control participants (females = 4; mean age = 46.50 years; SD age = 18.69 years; mean education = 16.13 years; SD education = 1.89 years; Table 2) were recruited. The visual impairment of the patient participants consisted of left homonymous hemianopia (*N* = 2), right homonymous hemianopia (*N* = 2), left homonymous inferior quadrantanopia (*N* = 3), and right homonymous superior quadrantanopia (*N* = 1; Table 1; Supplementary Fig. 1). Four additional patient participants were recruited but they were not included in analyses due to low data sample size (two participants), poor behavioral performance (one participant made incorrect keypresses and did not maintain central fixation), and not meeting the definition of cerebral blindness (one participant experienced visual impairment due to a chiasmal tumor).

The patient participants were recruited from the National Institute of Neurological Disorders and Stroke in Bethesda, Maryland (MD), USA and MedStar Georgetown University Hospital in Washington, District of Columbia, USA. Control participants were recruited from the local Bethesda, MD, USA community. All participants were recruited, consented, and tested in accordance with protocols approved by the Institutional Review Board of the National Institute of Mental Health. All ethical regulations relevant to human research participants were followed.

All patient participants completed two behavioral study sessions, except for participant P6 who completed only one session because they were lost to follow-up. In addition, to study the relationship between eye metrics and neural activity, participant P4 completed an additional magnetencephalography (MEG) study session (see *Magnetencephalography* section). MEG data were acquired only from P4 because the other patient participants were either ineligible (e.g., metal implanted in the body or large head size) or unable to participate due to limited testing availability and challenges traveling to the research facility, particularly for those with motor impairments. All control participants completed one behavioral study session. Fewer total trials were required for control participants because the left and right visual field stimulus presentation locations (see *Visual Perception Task* section) were combined in analyses, as both locations were sighted in control participants.

#### Inclusion criteria included

(1) 18 years of age or older, (2) at least a high school education (12 years or more), (3) capacity to provide their own informed consent, and understand and cooperate with study procedures, (4) neurologically normal (control participants only), and (5) unilateral or bilateral focal lesions and at least three months post-lesion (patient participants only). The average injury duration relative to the date of participation in the current study was ~21 months (Table 1).

#### Exclusion criteria included

(1) Any neurologic or psychiatric disorder unrelated to the focal lesion (e.g., epilepsy and schizophrenia; patient participants only), (2) previous head injury (control participants only), (3) present or past (within six months) drug or alcohol abuse or addiction, and (4) radiation treatment to the brain during a three-month period prior to the experiment (patient participants only).

The cause of cerebral blindness in the patient participants included ischemic stroke, hemorrhagic stroke, and traumatic brain injury (Table 1). Etiologies included hypertension, diabetes, atrial fibrillation, vascular surgery, a skiing accident, and a fall. For example, patient participant P4 suffered an ischemic stroke in the left cortical hemisphere. A structural whole-brain magnetic resonance imaging (MRI) scan revealed a lesion in the left primary visual cortex ventral to the calcarine sulcus (see *Structural Magnetic Resonance Imaging* section; Fig. 1A). A Humphrey visual field (HVF) test revealed a right homonymous superior quadrantanopia in P4 (Fig. 1B; Supplementary Fig. 1). See Table 1 and Supplementary Fig. 1 for clinical details and HVF test results for all patient participants.

### Visual perception task

The visual perception task consisted of trials with three primary phases (Fig. 1C): (1) a pre-stimulus fixation period (jittered 3–5 s), (2) a stimulus presentation period (3 s), and (3) a post-stimulus response and fixation period (jittered 3–5 s). Across all task phases, a central fixation cross (a black plus sign; behavioral session: visual angle = 0.99  × 0.99*°*; MEG session: visual angle = 0.38 × 0.38*°*) continuously appeared on a blank, gray screen.

For patient participants P7 and P8 and their paired control participants C7 and C8, the fixation cross was positioned to the top-center of the behavioral display monitor to maximize the lower visual field (see *Equipment and Testing Facility* section). This adjustment was made because the blind field for these patient participants extended deep into the periphery and were inaccessible to an on-screen stimulus presentation location with central fixation. Control and patient participants completed ~10 7-min task blocks comprising 40 trials each. After each task block, participants were prompted to provide a verbal report indicating whether they saw or sensed the presence of any task stimuli in their sighted and blind field (Tables 3 and 4).

Following the initial fixation period, a visual stimulus appeared (behavioral session: visual angle = 6.51 × 6.51*°*; MEG session: visual angle = 5.19 × 5.19*°*). Participants were instructed to maintain their gaze on the fixation cross at all times and not to directly look at the peripheral stimuli. Stimuli appeared in one of two mirror-symmetric screen locations equidistant from fixation. For the patient participants, one stimulus location was positioned in their sighted field and the second stimulus location was positioned in their blind field. During each task trial, a single peripheral stimulus appeared in either location at random but in equal proportion within each block.

The sighted and blind field locations were determined through a pre-task stimulus location calibration phase, guided by HVF test results that were made available to the experimenters prior to each study session, except for P6 who completed the HVF test after their study session (Supplementary Fig. 1). During location calibration, a central fixation and two nonglare stimuli (see stimulus details below) appeared in mirror-symmetric peripheral locations. The experimenter manually adjusted these locations while the patient participants centrally fixated and verbally reported their conscious awareness of the on-screen stimuli. The final stimulus presentation locations were set when the patient participant reported that they perceived only the stimulus positioned in their sighted field, and not the stimulus positioned in their blind field. For P2 and P7, no on-screen location could be found where these participants reported a complete absence of conscious awareness during location calibration, thereby a deep blind field location was selected according to their HVF test results. The colored circles overlaid on the HVF test in Fig. 1B and Supplementary Fig. 1 approximates the stimulus size and the sighted (yellow; P4 MEG session: orange) and blind field (blue; P4 MEG session: green) stimulus presentation locations for each patient participant. For control participants, the location calibration phase was not conducted. Instead, they were tested using the same stimulus presentation locations as their paired patient participants (Table 2).

The visual perception task presented five visual stimuli: (1) *glare*, (2) *nonglare*, (3) *isoluminant*, (4) *white*, and (5) *red* (Fig. 1C). Each stimulus type was presented in equal proportion within each block and across stimulus presentation locations with trial order randomized. There were two main stimulus categories: (1) *target* and (2) *nontarget*.

The target was the red stimulus. In an early version of the task administered to participants P1 session 1, C1, and C2, there was only one type of target stimulus: a plus sign-oriented (+) image with a central white square and four surrounding red squares (Fig. 1C). The remaining participants and P1 session 2 completed an updated task version with *two* target stimulus types that appeared in equal proportion: (1) plus sign and (2) x-oriented target stimuli (Fig. 1C). Both target stimulus types were identical except the x-oriented target stimulus was rotated 45*°* relative to the plus sign-oriented target stimulus. The two-target task version was introduced to test the visual acuity of patient participants who reported conscious awareness for task stimuli in their blind field. In the one-target task version, participants were prompted to select a single key on a keyboard immediately upon perceiving the target stimulus, regardless of its location on screen. In the two-target task version, participants were prompted to select one of two keys upon perceiving the target stimulus, regardless of its location on screen. Each key corresponded with either the plus sign or x-oriented target stimulus. The key mapping with the target orientation type was counterbalanced across participants. For patient participants who reported experiencing degraded conscious vision or non-visual sensations in their blind field, they were encouraged to respond to these vaguely seen or felt stimuli and to make their best guess about the target stimulus orientation.

The nontarget stimuli consisted of the glare, nonglare, isoluminant, and white stimuli. The glare stimulus was a plus sign-oriented image with a central white square and four surrounding squares with a black-to-white gradient facing inward (i.e., the white portion of the gradient faced the central white square). The glare stimulus and similar versions of this image are reported to induce the illusory perception of glare or brightness^39,40^. The nonglare stimulus was identical to the glare stimulus except the surrounding black-to-white gradient squares were rotated and did not induce the perception of illusory brightness (Supplementary Fig. 2). The isoluminant stimulus was identical to the nonglare stimulus except the central white square was replaced with a gray square identical in luminance to the gray screen background. Importantly, the black-to-white gradient squares in the glare, nonglare, and isoluminant stimuli had an average luminance equal to the gray screen background. Therefore, the average total luminance of the isoluminant stimulus was equal to the gray screen background. The isoluminant stimulus was included to evaluate the influence of stimulus presentation on eye metrics independent of luminance change, as the other nontarget stimuli involved increased luminance relative to the gray screen background. Finally, the white stimulus appeared as an all-white version of the glare, nonglare, and isoluminant stimuli, serving as a physically bright control to maximally elicit the pupillary light response. For all nontarget stimuli, participants were instructed to withhold any response when they appeared, as they were task irrelevant.

For patient participant P4, the visual perception task was modified into two additional task versions: (1) behavioral-adapted and (2) MEG-adapted. The behavioral-adapted task maintained the trial structure as the original visual perception task (Fig. 1C), but presented only the target stimulus (i.e., plus sign and x-oriented red stimuli). P4 was instructed to make an immediate keypress whenever he perceived the target stimulus in his sighted field or experienced what he described as a vision or feeling of “movement” and “vibration” in his blind field (see *Visual perception task* Visual behavior Results section; Table 3). Similar to the original perception task, P4 was instructed to make a keypress to indicate the target orientation and, if uncertain, to make his best guess for its orientation. The stimulus presentation locations were identical to those used for P4 in the original visual perception task (Fig. 1B; Supplementary Fig. 1).

The MEG-adapted visual perception task maintained the trial structure as the original visual perception task (Fig. 1C), but included only the target stimuli and a subset of the nontarget stimuli: (1) glare and (2) nonglare stimuli. This change was made to increase the number of trials for these stimulus types. Also, because P4 suffered from right homonymous superior quadrantanopia (upper right quadrant vision loss), the sighted stimulus position was adjusted from the behavioral task to appear in the sighted, bottom right quadrant of the visual field (Supplementary Fig. 1). This adjustment was made so that the MEG field potentials for stimuli presented in the sighted and blind field, now both located in the right visual field, would correspond with contralateral field potential changes in the *left* visual cortex.

### Brightness perception task

Previous studies report that the glare stimulus induces a percept of illusory brightness^39,40^. To gauge if the participants in the current experiment also perceived illusory brightness from the glare stimulus, participants completed a brightness perception task (Supplementary Fig. 2A). Each task trial began with a blank gray screen (2 s), followed by a side-by-side presentation of two stimuli among the glare, nonglare, and isoluminant stimuli (see *Visual Perception Task* section for stimulus details). Participants were instructed to report with keypresses which stimulus appeared brighter near its center or if both images appeared with equal brightness. Participants could look at the images directly and their responses were self-paced. Participants completed a single 30-trial task block with 10 trials each of the following stimulus comparisons: (1) glare versus nonglare, (2) glare versus isoluminant, and (3) nonglare versus isoluminant stimulus. The on-screen stimulus presentation locations were identical for all participants and the patient participants reported being able to clearly see the stimuli prior to making a response because they were able to directly fixate on each stimulus.

### Pupillometry and eye tracking

Head-fixed (SR Research Head Support; SR Research, Inc.) monocular pupillometry and eye tracking were acquired with the EyeLink 1000 Plus (sampling rate = 1000 Hz; SR Research, Inc.). Whichever eye was best positioned with the eye tracker camera was selected for recording (Tables 1 and 2). The EyeLink 1000 Plus software and monitoring of eye tracking during the study session was performed on a Dell OptiPlex XE2 desktop computer and monitor (Dell, Inc.). The behavioral computer (see *Testing Equipment and Facility* section) and EyeLink desktop communicated via an Ethernet connection. Participants were positioned ~56 and 106 cm from the EyeLink camera in the behavioral and MEG study sessions, respectively.

### Magnetencephalography

MEG data were recorded from patient participant P4 using a CTF 275 MEG system (sampling rate = 1200 Hz; CTF Systems, Inc., Canada) composed of a whole-head array of 275 radial 1st order gradiometer sensors housed in a magnetically shielded room (Vacuumschmelze GmbH & Co. KG, Germany). Data were not recorded from three malfunctioning sensors, including one right occipital sensor (O13; Supplementary Fig. 7B). Synthetic 3rd gradient balancing was used to remove background noise. The MEG acquisition software was run on a Dell Precision T7500 desktop computer (Dell, Inc.). While recording the MEG data, P4 completed the MEG-adapted visual perception task (see *Visual Perception Task* section). Behavioral task events (e.g., stimulus onset) were marked in the MEG recording via parallel port. Simultaneously, head-fixed pupillometry and eye tracking were recorded with the EyeLink 1000 Plus system (SR Research, Inc.; see *Pupillometry and Eye Tracking* section).

### Structural magnetic resonance imaging

A whole brain structural T1-weighted MRI (magnetization prepared—rapid gradient echo [MPRAGE]) was acquired from patient participant P4 with a 3T MR750 MRI (General Electric, Inc.) and a 32-channel head coil (Nova Medical, Inc.).

### Testing equipment and facility

All experimental sessions were completed in a windowless, temperature-controlled room at the National Institutes of Health, Bethesda, MD, USA. Each study session lasted ~2.5 hours, including a health exam, instructions, and task breaks. During the behavioral session, the experimenters were positioned behind the participant to monitor behavior and deliver task instructions. During the MEG session, the experimenters were positioned outside the MEG shielded room and monitored and communicated with the participant via a closed-circuit television (COLOR CCD Camera VCC-3912; Sanyo Electric Co. Ltd) and intercom console system (VSM MedTech Ltd).

The behavioral tasks (see *Visual Perception Task* and *Brightness Perception Task* sections) were coded in Python and run with PsychoPy (behavioral session: v2022.2.4; MEG session: v2021.1.2; Open Science Tools Ltd). During the behavioral study sessions, the tasks were run on a laptop (MacBook Pro 2019; 13-inch; 2560 × 1600 pixels; Mac OS Catalina v10.15.7; Apple, Inc.) and participants viewed the behavioral tasks on a VPixx monitor (1920 × 1200 pixels; VPixx Technologies, Inc.) that mirrored the laptop display via a DVI connection. During the MEG study session, the behavioral task was run on a Dell Precision T3500 desktop computer (Dell, Inc.). Patient participant P4 viewed the task on a projector screen that mirrored the desktop display via a PROPixx LED projector (VPixx Technologies, Inc.) positioned outside the MEG shielded room. The projected light first passed through a waveguide, then an adjustable two-mirror system that reflected the display image onto the projection screen. Participants were positioned ~58 and 75 cm from the behavioral monitor and MEG projector screen, respectively.

For all study sessions, participants were instructed to make their responses during the behavioral task with their right hand. During the behavioral study session, participants made keypresses using a keyboard positioned on a table in front of them. During the MEG study session, P4 made button presses using a button box (4 Button Inline Fiber Optic Response Pad; Current Designs, Inc.). Button presses were received via an electronic interface (932 Interface & Power Supply; Current Designs, Inc.) and marked in dedicated channels of the MEG recordings.

### Statistics and reproducibility

All analyses were completed in MATLAB (R2023b; Mathworks, Inc.). Data and results were visualized using AFNI (version 25.1.07)^50,51^, MATLAB (R2023b; Mathworks, Inc.), Prism (version 10.4.0; GraphPad Software, LLC.), and Illustrator (Adobe, Inc.).

### Visual perception task

Perception rate and orientation discrimination accuracy were calculated for the target stimulus (i.e., the red stimuli; see *Visual Perception Task* section; Figs. 2 and 6B, C, and E). Note that perception rate and accuracy rate could not be calculated for the nontarget stimuli because participants were not instructed to respond to them (i.e., nontarget stimuli were task irrelevant). *Perception rate* was calculated as the number of *perceived* target stimuli divided by the total number of *presented* (perceived + not perceived) target stimuli. A target stimulus was considered perceived if the participant responded with a key or button press within 5000 ms from the onset of the target stimulus, regardless of orientation accuracy. *Orientation discrimination accuracy* was calculated as the number of *correctly identified* target orientations (see *Visual Perception Task* section) divided by the total number of *perceived* target stimuli. Chance level target orientation accuracy was 0.5.

For control participants, perception rate and accuracy were calculated separately within the *left* and *right* visual field stimulus presentation locations (Fig. 2A and C). For patient participants, perception rate and accuracy were calculated separately within the *sighted* and *blind* field stimulus presentation locations (Fig. 2B and D). Accuracy could not be calculated for C1 and C2 who completed the one-target visual perception task version (see *Visual Perception Task* section). Blind field accuracy was calculated for only patient participants P2 and P7 who reported high (>0.9) blind field perception rate (open circles in Fig. 2B; see *Behavior* Results section). The remaining patient participants reported low (<0.1) blind field perception rate (closed circles in Fig. 2B; see *Behavior* Results section), resulting in too few perceived target stimulus trials to reliably calculate accuracy. Left versus right field (control participants) and sighted versus blind field (patient participants) perception rate and accuracy were statistically compared with Wilcoxon matched-pairs signed-rank tests with Holm-Bonferroni correction to control for multiple comparisons. Sighted versus blind field (patient participants) accuracy was not statistically evaluated.

Additional behavioral analyses were conducted on P4’s performance in the behavioral-adapted visual perception task. First, the false positive rate was defined as the number of keypresses occurring *more than* 5000 ms after the onset of a target stimulus (i.e., keypresses not temporally associated with stimulus presentations) divided by the total number of keypresses. Also, P4’s keypress reaction times relative to stimulus onset were statistically compared between the sighted and blind field with a Wilcoxon matched-pairs signed-rank test (Fig. 6D). Finally, to assess whether P4’s target orientation discrimination accuracy was statistically greater than chance, a one-sided binomial test was conducted (Fig. 6E).

### Brightness perception task

Perceived brightness of the glare, nonglare, and isoluminant stimuli was calculated using a custom scoring system: for each trial that the participant reported a stimulus as brighter than the juxtaposed image, the stimulus perceived as brighter received 1 point; 0.5 points if the stimulus was reported as equally bright; and 0 points if the stimulus was reported as less bright. Participants completed 10 trials for each of the following stimulus pairings: (1) glare versus nonglare, (2) glare versus isoluminant, and (3) nonglare versus isoluminant stimulus. Thereby, a stimulus that was always reported as brighter would receive a maximum score of 20, while a stimulus that was always reported as dimmer would receive a minimum score of 0. Within-participant brightness perception reports were tested with a Friedman test and post-hoc Wilcoxon matched-pairs signed-rank tests with Holm-Bonferroni correction to control for multiple comparisons (glare versus nonglare and nonglare versus isoluminant stimulus; Supplementary Fig. 2B and C).

### Eye metrics

#### Pupil size epoch extraction

The pupil data were preprocessed, including the removal of blinks and smoothing^52^. The preprocessed pupil data were segmented into 18,001-ms epochs centered at stimulus onset. Also, interstimulus interval or blank event epochs were segmented for each stimulus type, centered between 4000 and 7000 ms after stimulus onset (i.e., a minimum of 1000 ms after the offset of the preceding stimulus and before the onset of the subsequent stimulus). The blank event time was calculated by selecting a random time within the interstimulus interval following each stimulus and preceding the subsequent stimulus or task block end time (for the final trial blank event in each task block). Therefore, each stimulus epoch had a corresponding blank event epoch. Finally, all stimulus and blank event epochs were baselined to the average pupil size within the 1000 ms immediately preceding the stimulus onset or blank event. Pupil size epochs were excluded from analysis if between 1000 ms pre-stimulus onset or blank event and 6000 ms post stimulus onset or blank event there was an extreme pupil size value (>1750 pixels; threshold selected by visual inspection of the pupil size epoch timecourses) or if more than 50% of the samples within this epoch interval did not have a pupil size value (e.g., due to prolonged eye closure or loss of eye tracking). Across all subjects and events, these exclusion criteria resulted in the removal of less than 15% of pupil size epochs. Finally, epochs were averaged within participant across event type (e.g., the nontarget condition results were an average of the glare, nonglare, isoluminant, and white stimulus epochs; Fig. 3) and stimulus location.

#### Blink epoch extraction

Blinks were determined by the pupil size preprocessing method that identified blink intervals (see *Pupil size epoch extraction* section). In summary, blinks were defined using multiple independent criteria, including rapid fluctuations in pupil size and the presence of outlier values in the pupil size data. The resulting blink data set was a binary timecourse (0 = blink absent; 1 = blink present) with an equal number of samples as the pupil size data. Blink epochs were segmented centered at stimulus and blank event onset times exactly as specified for the pupil size epochs (see *Pupil size epoch extraction* section), except that no baselining was performed on the blink epochs. Blink epochs were removed from analysis if more than 50% of the epoch samples between 1000 ms pre-stimulus onset or blank event and 6000 ms post stimulus onset or blank were 0 (e.g., due to prolonged eye closure or loss of eye tracking). This epoch removal criterion resulted in the removal of less than 15% of blink epochs. Finally, epochs were averaged within participant across event type and stimulus presentation location and smoothed. The resulting mean blink fraction timecourses indicated the proportion of trials that a blink event was present for each epoch sample.

#### Microsaccade epoch extraction

Microsaccade events were determined from the gaze position data acquired simultaneously with pupillometry^53^. In summary, microsaccades were identified when the velocity of horizontal and vertical eye movements exceeded median-based thresholds, as validated in ref. ^54^. The resulting microsaccade data set was a binary timecourse (0 = microsaccade absent; 1 = microsaccade present) with an equal number of samples as the pupil size data. Microsaccade epochs were segmented centered at the stimulus onset and blank event times exactly as specified for the blink fraction epochs (see *Blink epoch extraction* section). Also, the same blink epoch removal criterion was applied to the microsaccade epochs resulting in removing fewer than 15% of microsaccade epochs. Finally, remaining epochs were averaged within participant by event type and stimulus presentation location and smoothed. The resulting mean microsaccade fraction timecourses indicated the proportion of trials that a microsaccade event was present for each epoch sample.

#### Eye metric visualization and statistical analysis

For control participants, eye metric epoch timecourses (pupil size, blink, and microsaccade) were averaged between the left and right visual field stimulus presentation locations (Figs. 3A and 5A; Supplementary Fig. 3A). For patient participants, the eye metric epoch timecourses were averaged separately within the sighted and blind field stimulus presentation locations (Figs. 3B, C, and D, 5C and E, and 6A; Supplementary Fig. 3B, C, and D; Supplementary Fig. 6). The blind field responses were visualized and statistically evaluated independently between blind aware and blind unaware patient participants (Figs. 3C and D, and 5E; Supplementary Fig. 3C and D; see *Visual perception task* Visual behavior Results section for the definitions of blind aware and unaware patient participants).

To evaluate pupil size responses to real and illusory luminance, pupillary constriction was compared across the white, glare, and nonglare stimuli (Fig. 5B, D, and F). For each participant, the *minimum* pupil size within the first 1500 ms following stimulus onset was extracted from their mean pupil timecourses (the analysis window represented by the horizontal dotted line in Fig. 5A, C, and E). A linear regression (first-degree polynomial) was fit to each participant’s minimum pupil size values across the three stimulus types, ordered as white, glare, and nonglare stimuli. To assess the overall trend, the fitted slopes were tested across participants against a null slope of 0 using Wilcoxon matched-sign rank sum tests. A significant positive slope would suggest the expected pattern of pupil modulation: the largest constriction for the white stimulus, intermediate for the glare stimulus, and weakest for the nonglare stimulus, consistent with previous reports of real and illusory luminance effects (e.g.,)^45^. Pupillary constriction in the blind field of blind unaware patient participants was not analyzed, as these individuals did not exhibit a measurable stimulus-evoked pupillary response.

#### Eye metric-based classification

Epoch-level, stimulus-evoked eye metric responses were evaluated using participant-level classifiers trained on the pupil size, blink, and microsaccade epoch data. *Four* sets of classifiers were trained and tested for each participant. For control participants, the trained classifiers were: (1) left and (2) right visual field *target stimulus* versus blank event, and (3) left and (4) right visual field *nontarget stimulus* versus blank event. For patient participants, the trained classifiers were: (1) sighted and (2) blind field *target stimulus* versus blank event, and (3) sighted and (4) blind field *nontarget stimulus* versus blank event. In summary, all classifiers were trained to predict a stimulus versus blank event epoch based on eye metric responses.

A two-step, stacking classification approach was implemented to assess stimulus-evoked eye metrics on an epoch-by-epoch basis (Supplementary Fig. 4). First, pupil size, blink, and microsaccade linear support vector machine classifiers (i.e., three independent classifiers for each eye metric) were trained using tenfold cross-validation on epoch samples within 4000 ms post stimulus onset. Next, the predicted scores (i.e., the signed distance from the decision boundary) for each epoch from the first-level pupil size, blink, and microsaccade classifiers were used as features in a second-level, ensemble linear support vector machine classifier using the same cross-validation folds as in the first-level classifier (i.e., nested cross-validation) to prevent data leakage between the first and second-level classifiers. Finally, the predicted epoch classes from the second-level classifier were used to assess classification performance. *Prediction accuracy* was calculated as the number of *correctly* predicted epochs divided by the number of *correctly* and *incorrectly* predicted epochs (Fig. 4; Supplementary Fig. 5). Chance level accuracy (i.e., the proportion of trials of a given type relative to the total number of trials) was calculated for each participant and stimulus presentation location condition.

In group-level statistical analyses, Wilcoxon matched-pairs signed-rank tests evaluated whether accuracy was statistically *greater* than chance. Additionally, Wilcoxon matched-pairs signed-rank tests were conducted to determine whether accuracy differed between the left and right visual fields in control participants, and between the sighted versus blind fields in patient participants. Finally, Mann–Whitney U tests evaluated if the accuracy was statistically *greater* in the blind field for blind aware versus blind unaware patient participants. Holm-Bonferroni correction was applied separately to the set of analyses testing against chance and cross-conditions (e.g., sighted versus blind field) comparisons to control for multiple comparisons.

### Correspondence among blind field task behavior, verbal perceptual report, and eye metric responses

Three main measures were acquired from all patient participants: (1) behavioral performance on the visual perception task, (2) verbal report on perceptual experiences related to stimulus presentation during the visual perception task, and (3) stimulus-evoked eye metric responses. Evaluating the correspondence among these measures could help assess their interactions and robustness, particularly for indicating blind field visual conscious perception. For patient participants, each measure was evaluated as *present* or *absent* in the blind field. *Task behavior* was designated *present* if the target stimulus perception rate was >0.25. Verbal report indicative of blind field conscious awareness was designated *present* if conscious vision, including degraded abnormal vision and non-visual sensations were reported for >10% of task stimuli. Finally, stimulus-evoked eye metric responses were designated *present* in the blind field if stimulus evoked responses were observed in at least one of the eye metrics (pupil size, blink, or microsaccade; e.g., Fig. 6A; Supplementary Fig. 6).

### MEG

MEG analyses were completed using custom functions and the FieldTrip toolbox (http://fieldtriptoolbox.org)^55^; First, the MEG data were preprocessed on a sensor basis, including applying bandpass filtering (0.1 and 115 Hz) and line noise removal (60 and 120 Hz). Next, the preprocessed MEG data were segmented into 8001-ms epochs centered at stimulus onset and blank event times (see *Visual Perception Task* section). Stimulus event times were determined from parallel port triggers recorded simultaneously with MEG sensors. An event time correction of 19 ms was applied to the trigger onset times to correct for the delay in the presentation of task stimuli on the projector screen (see *Testing Equipment and Facility* section). The blank event times were determined using the same method specified for pupil size blank event epochs (see *Pupil epoch extraction* section).

Sensor-level field potential averages and standard error of the mean across epochs were calculated within event type and stimulus presentation locations (sighted versus blind visual field). The MEG results reported in Fig. 6F and Supplementary Fig. 7 represent the average field potential across all nontarget stimuli (glare and nonglare stimuli; see MEG-adapted visual perception task details in the *Visual perception task* Methods section) in the sighted (yellow) and blind field (blue), while the blank event timecourse (black) represents an average between the sighted and blind field.

Sensor-evoked changes in field potential among the sighted nontarget, blind nontarget, and sighted + blind field blank event conditions were assessed using cluster-based permutation testing (5000 permutations)^56^; The permutation analysis baseline interval was 500 ms preceding the stimulus onset or blank event. The 500 ms following the stimulus onset or blank event were evaluated for statistically significant samples. Three statistical comparisons were evaluated: (1) sighted nontarget stimuli versus sighted field blank events, (2) blind nontarget stimuli versus blind field blank events, and (3) sighted nontarget stimuli versus blind field nontarget stimuli (Fig. 6F). Statistical testing was not performed on the sensors depicted in Supplementary Fig. 7.

### Reporting summary

Further information on research design is available in the Nature Portfolio Reporting Summary linked to this article.

## Supplementary information

Supplementary Information

Reporting Summary

## Data Availability

Source data are available at https://osf.io/cygmj/.

## Code availability

Analysis codes are available at https://github.com/nimh-sfim/Eye-Metrics-Cerebral-Blindness.
