# Supplementary material for: Eye metrics often reflect visual conscious awareness, conscious content, and neural processing in cerebral blindness
Source: Commun Biol. 2025 Dec 1;8:1724. doi: 10.1038/s42003-025-08945-5 (PMC12669680; doi:10.1038/s42003-025-08945-5)
Supplement: Supplementary file 1 — Supplementary Information [file 42003_2025_8945_MOESM1_ESM.pdf]

Supplementary Information

Eye metrics often reflect visual conscious awareness, conscious content, and neural processing in cerebral blindness

Sharif I. Kronemer[1], Victoria E. Gobo[1], Shruti Japee[1], Elisha P. Merriam[1], Benjamin Osborne[2], Peter A. Bandettini[1,3], Tina T. Liu[1,4]

Affiliations

[1]Laboratory of Brain and Cognition (LBC), National Institute of Mental Health (NIMH), National Institutes of Health (NIH), Bethesda, Maryland (MD), USA

[2] Department of Neurology and Ophthalmology, Medstar Georgetown University Hospital, Washington, District of Columbia (DC), USA

[3] Functional MRI Facility, NIMH, NIH, Bethesda, MD, USA

[4] Department of Neurology, Georgetown University Medical Center, Washington, DC, USA

Corresponding Author: Sharif I. Kronemer (sharif.kronemer@nih.gov)

Contents

Supplementary Figures 1-7

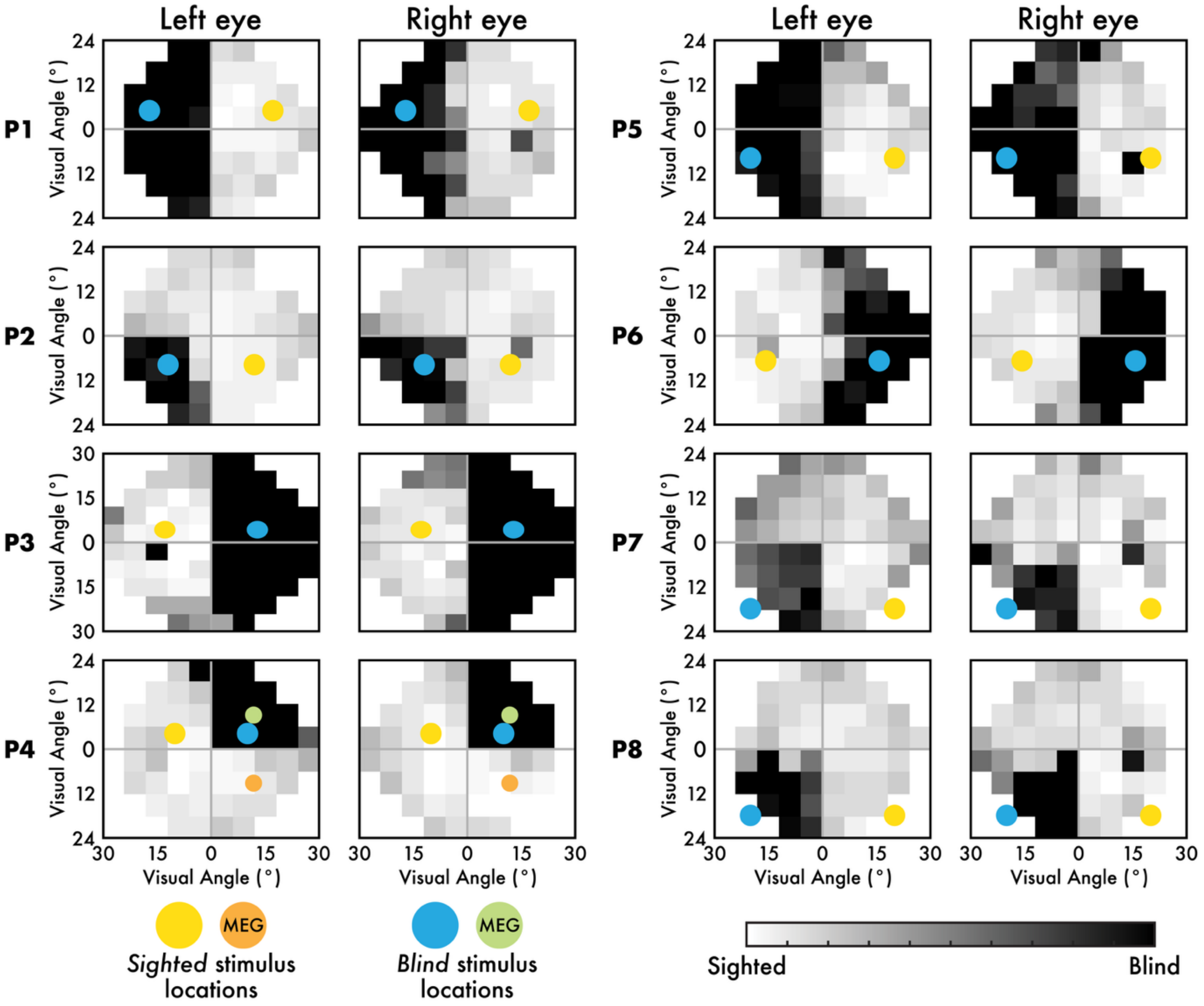

Supplementary Figure 1. *Humphrey visual field test results and sighted and blind visual field stimulus presentation locations*. The Humphrey visual field (HVF) test results are shown for the left and right eyes in degrees (°) of visual angle. Sighted areas are indicated by white and light gray squares, while blind areas are indicated by dark gray and black squares. The single black or dark gray square present for most patient participants in the left visual field (left eye) or the right visual field (right eye) is the natural blind spot. The colored circles depict the approximate size and location of the visual perception task stimuli shown in the sighted (yellow; MEG study session: orange) and blind field (blue; MEG study session: green).

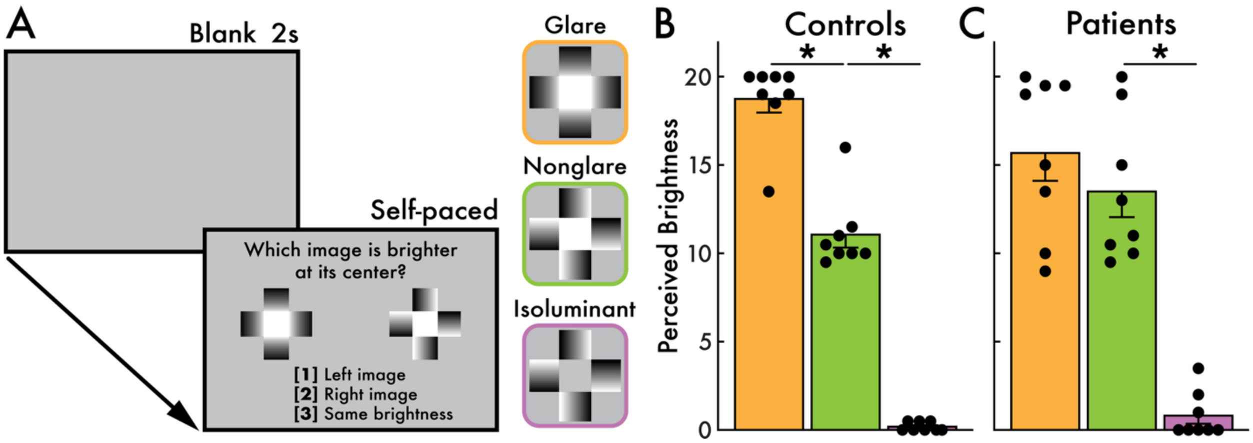

Supplementary Figure 2. *Brightness perception task and behavioral results*. **(A)** Brightness perception task trial structure (see *Brightness Perception Task* Methods section for full details). First, a 2-second (s) blank interval appeared. Next, participants were shown two stimuli side-by-side among three possible stimulus types: (1) glare, (2) nonglare, and (3) isoluminant stimulus. Participants were prompted to answer the question "Which image is brighter at its center?". Participants could view these stimuli directly and for an unlimited duration. Participants selected the 1-key if the left image was perceived as brighter, the 2-key if the right image was perceived as brighter, and the 3-key if both images appeared with equal brightness. Once the participant made their response, a new trial would begin with an updated image comparison pair. Participants completed 30 trials total with 10 trials each of the following stimulus pairs: (1) glare versus nonglare, (2) glare versus isoluminant, and (3) nonglare versus isoluminant stimulus. **(B)** Perceived brightness for the glare, nonglare, and isoluminant stimuli in control participants (N = 8). Larger values (maximum = 20; minimum = 0) indicated that participants perceived that stimulus as brighter. The glare stimulus was reported as significantly brighter than the nonglare stimulus, and the nonglare stimulus was reported as significantly brighter than the isoluminant stimulus (*; $p < 0.05$). **(C)** Perceived brightness for the glare, nonglare, and isoluminant stimuli in patient participants (N = 8). Most patient participants reported that the glare stimulus was brighter than the nonglare stimulus, however, this response trend was not statistically significant. The nonglare stimulus was reported as significantly brighter than the isoluminant stimulus. In B and C, the circles represent individual participants, and the bars and error bars indicate the group mean and standard error of the mean, respectively.

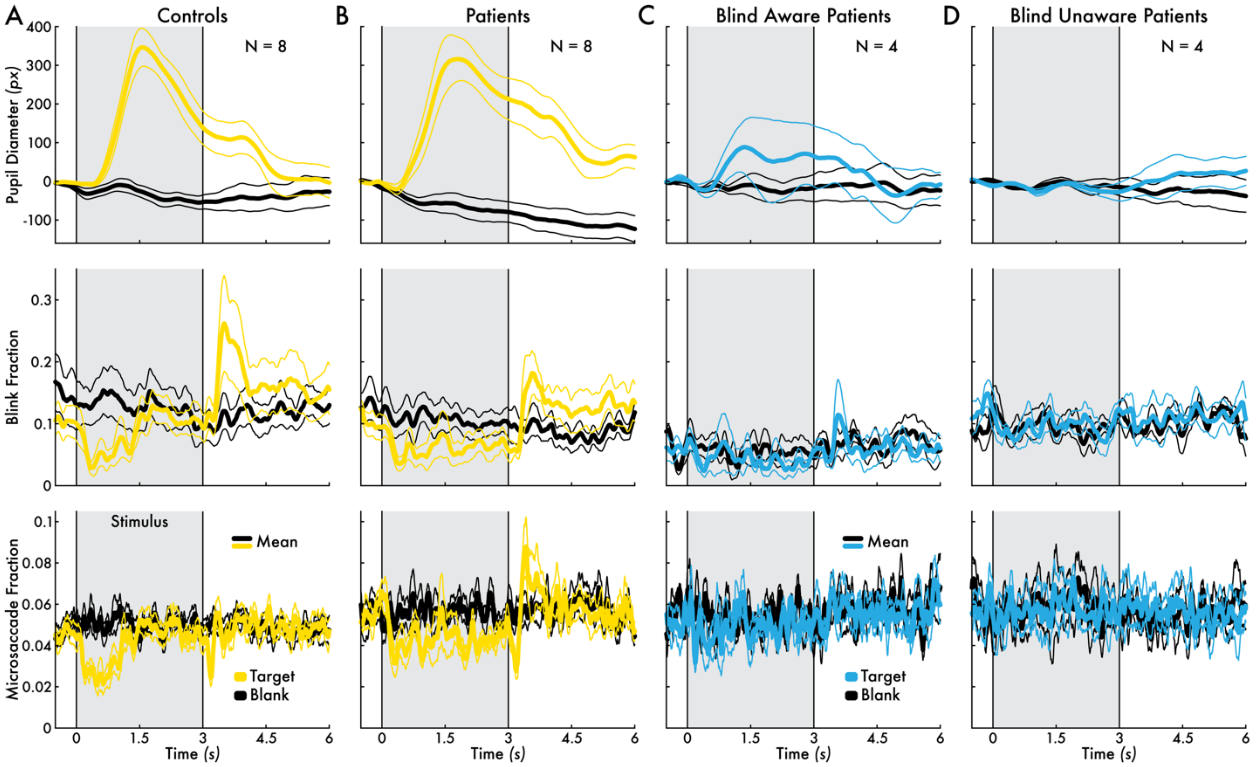

Supplementary Figure 3. *Target stimulus-evoked pupil, blink, and microsaccade responses*. Pupil diameter, blink fraction, and microsaccade fraction change preceding (0.5 seconds [s]) and following (6 s) the target stimulus (averaged between the plus sign and x-oriented target stimuli; see *Visual Perception Task* Methods section; yellow = sighted visual field; blue = blind visual field) for **(A)** control participants (N = 8) averaged between the left and right visual fields, **(B)** patient participants (N = 8) sighted field, **(C)** blind aware patient participants (N = 4) blind field, and **(D)** blind unaware patient participants (N = 4) blind field. For all subplots, corresponding blank event pupil diameter, blink fraction, and microsaccade fraction responses are shown (black). The group mean eye metric timecourses are shown (thicker traces) bounded by the standard error of the mean (thinner traces). The 3-s stimulus presentation interval is highlighted by a gray area bounded between two vertical lines at 0 and 3 s.

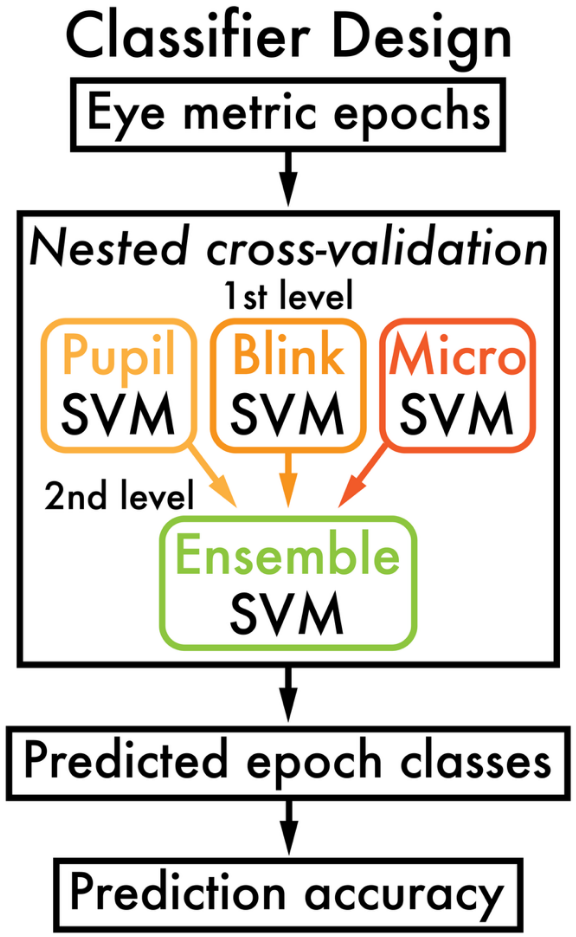

Supplementary Figure 4. *Schematic of the two-step stacking classifier design*. In the first level, linear support vector machine (SVM) classifiers for pupil, blink, and microsaccade (micro) data were trained to predict target or nontarget stimulus versus blank event epochs. The predicted scores from these first-level classifiers were then used as features to train a second-level, ensemble linear SVM, which was also trained to predict target or nontarget stimulus versus blank event epochs. A nested 10-fold cross validation approach was implemented to prevent data leakage between the first and second-level classifiers. Finally, the predicted epoch classes from the ensemble SVM were used to assess the accuracy of epoch class predictions.

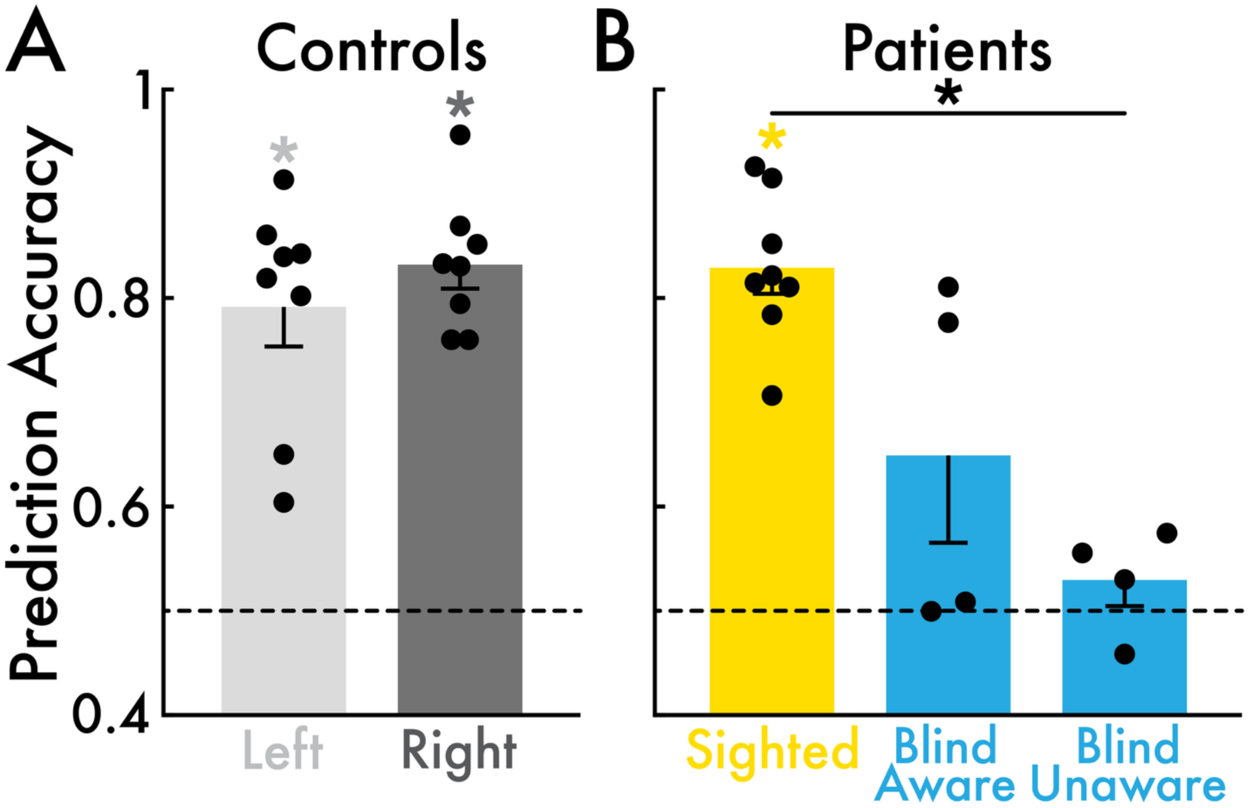

Supplementary Figure 5. *Eye metric-based target stimulus versus blank event classification performance.* **(A)** Prediction accuracy for the left (light gray) and right (dark gray) visual fields in control participants (N = 8). Left and right visual fields accuracy were significantly greater than chance (*; $p < 0.05$). Left versus right visual field accuracy were not significantly different. **(B)** Prediction accuracy for the sighted (yellow) and blind fields (blue) in patient participants (N = 8; blind aware patient participants: N = 4; blind unaware patient participants: N = 4). Accuracy was significantly greater than chance for the sighted field but not significant for the blind field for the blind aware and unaware participants. Sighted field accuracy was significantly greater than the blind field. In all subplots, chance level was approximately 0.5 (highlighted with a dotted line), the circles represent individual participants, and the bars and error bars indicate the group mean and standard error of the mean, respectively.

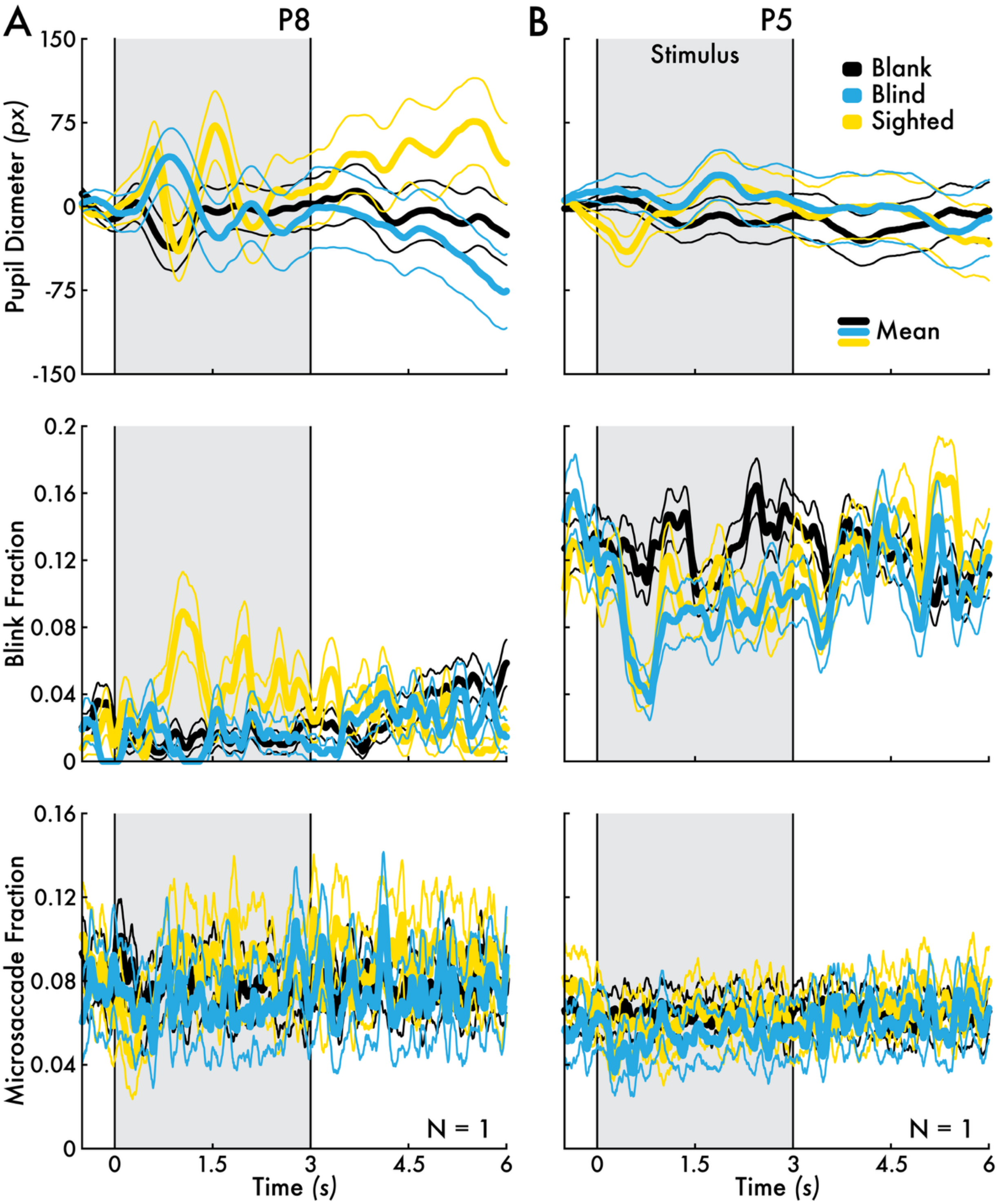

Supplementary Figure 6. *Patient participants P8 and P5 sighted and blind visual field stimulus-evoked eye metric responses for nontarget stimuli.* Patient participant **(A)** P8 and **(B)** P5 sighted (yellow) and blind visual fields (blue) pupil diameter, blink fraction, and microsaccade fraction change for nontarget stimuli (averaged across the white, glare, nonglare, and isoluminant stimuli) versus blank events (black). The mean eye metric changes across all nontarget stimulus epochs are shown (thicker traces) bounded by the standard error of the mean (thinner traces).

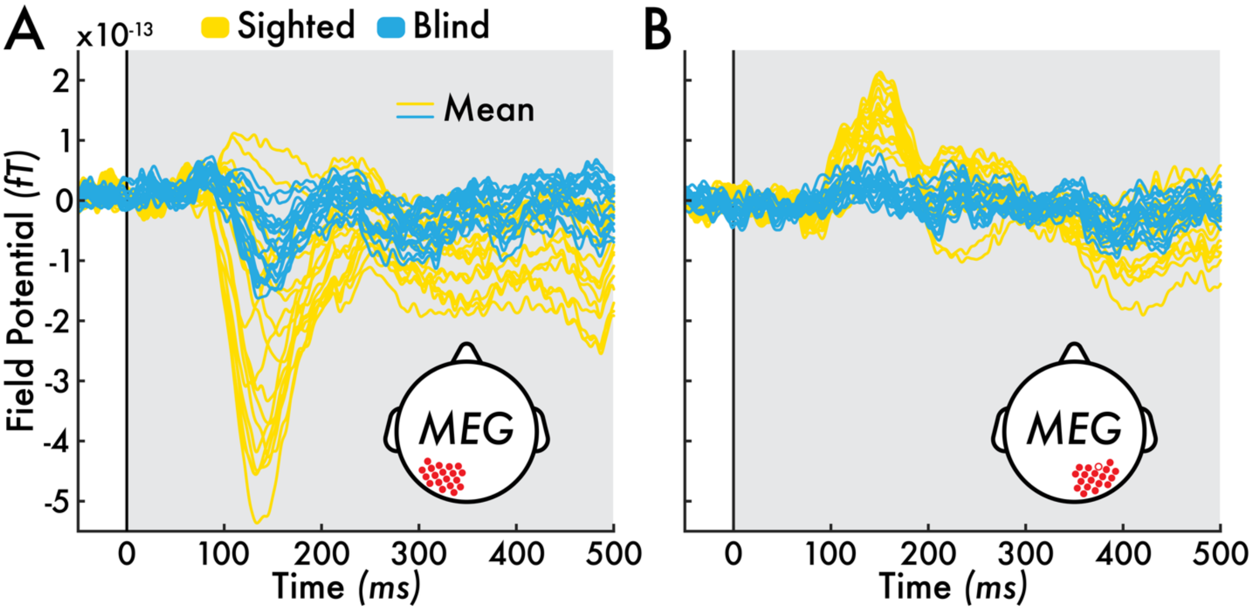

Supplementary Figure 7. *Patient participant P4 sighted and blind visual fields MEG responses for nontarget stimuli.* The mean nontarget stimulus-evoked magnetencephalography (MEG) field potentials (femtotesla; fT) up to 500 milliseconds (ms) from the target stimulus presentation onset in the sighted (yellow) and blind visual fields (blue) for **(A)** left (sensors = 19) and **(B)** right occipital sensors (sensors = 18; see inset diagram for approximate sensor locations on the scalp; malfunctioning right occipital sensor O13 was not recorded; highlighted with an open circle). The MEG results were acquired with the MEG-adapted visual perception task (see *Visual Perception Task* Methods section). The stimulus presentation onset is indicated by a vertical line at 0 ms and the stimulus presentation interval is highlighted by a gray area.
