# Supplementary material for: Eye metrics often reflect visual conscious awareness, conscious content, and neural processing in cerebral blindness
Source: Commun Biol. 2025 Dec 1;8:1724. doi: 10.1038/s42003-025-08945-5 (PMC12669680; doi:10.1038/s42003-025-08945-5)
Supplement: Supplementary file 2 — Reporting Summary [file 42003_2025_8945_MOESM2_ESM.pdf]

nature portfolio

Corresponding author(s): Sharif I. Kronemer

Last updated by author(s): 2025/09/12

Reporting Summary

Nature Portfolio wishes to improve the reproducibility of the work that we publish. This form provides structure for consistency and transparency in reporting. For further information on Nature Portfolio policies, see our Editorial Policies and the Editorial Policy Checklist.

Statistics

For all statistical analyses, confirm that the following items are present in the figure legend, table legend, main text, or Methods section.

n/a	Confirmed	
☐	☒	The exact sample size (*n*) for each experimental group/condition, given as a discrete number and unit of measurement
☐	☒	A statement on whether measurements were taken from distinct samples or whether the same sample was measured repeatedly
☐	☒	The statistical test(s) used AND whether they are one- or two-sided<br>*Only common tests should be described solely by name; describe more complex techniques in the Methods section.*
☐	☒	A description of all covariates tested
☐	☒	A description of any assumptions or corrections, such as tests of normality and adjustment for multiple comparisons
☐	☒	A full description of the statistical parameters including central tendency (e.g. means) or other basic estimates (e.g. regression coefficient) AND variation (e.g. standard deviation) or associated estimates of uncertainty (e.g. confidence intervals)
☐	☒	For null hypothesis testing, the test statistic (e.g. *F*, *t*, *r*) with confidence intervals, effect sizes, degrees of freedom and *P* value noted<br>*Give P values as exact values whenever suitable.*
☒	☐	For Bayesian analysis, information on the choice of priors and Markov chain Monte Carlo settings
☒	☐	For hierarchical and complex designs, identification of the appropriate level for tests and full reporting of outcomes
☒	☐	Estimates of effect sizes (e.g. Cohen's *d*, Pearson's *r*), indicating how they were calculated

Our web collection on statistics for biologists contains articles on many of the points above.

Software and code

Policy information about availability of computer code

Data collection	PsychoPy (behavioral session: v2022.2.4; MEG session: v2021.1.2; Open Science Tools Ltd.)
Data analysis	MATLAB (R2023b; Mathworks, Inc.); Prism (version 10.4.0; GraphPad Software, LLC.)

For manuscripts utilizing custom algorithms or software that are central to the research but not yet described in published literature, software must be made available to editors and reviewers. We strongly encourage code deposition in a community repository (e.g. GitHub). See the Nature Portfolio guidelines for submitting code & software for further information.

Data

Policy information about availability of data

All manuscripts must include a data availability statement. This statement should provide the following information, where applicable:

- Accession codes, unique identifiers, or web links for publicly available datasets
- A description of any restrictions on data availability
- For clinical datasets or third party data, please ensure that the statement adheres to our policy

Source data are available at https://osf.io/cygmj/. Analysis codes are available at https://github.com/nimh-sfim/Eye-Metrics-Cerebral-Blindness.

Research involving human participants, their data, or biological material

Policy information about studies with human participants or human data. See also policy information about sex, gender (identity/presentation), and sexual orientation and race, ethnicity and racism.

Reporting on sex and gender	Findings do not apply to only one sex or gender and was not considered in the study design. Sex was determined based on self-reporting. Sex-based analyses were not performed due to insufficient statistical power.
Reporting on race, ethnicity, or other socially relevant groupings	Ethnicity was determined based on self-reporting. Other socially relevant groupings were not inquired. Ethnicity-based analyses were not performed due to insufficient statistical power.
Population characteristics	Healthy adult participants and cerebrally blind participants were tested in this study. Both participant groups were age and education matched.
Recruitment	The patient participants were recruited from the National Institute of Neurological Disorders and Stroke in Bethesda, Maryland (MD), USA and MedStar Georgetown University Hospital in Washington, District of Columbia, USA. Control participants were recruited from the local Bethesda, MD, USA community.
Ethics oversight	The Institutional Review Board of the National Institute of Mental Health

Note that full information on the approval of the study protocol must also be provided in the manuscript.

Field-specific reporting

Please select the one below that is the best fit for your research. If you are not sure, read the appropriate sections before making your selection.

☐ Life sciences ☒ Behavioural & social sciences ☐ Ecological, evolutionary & environmental sciences

For a reference copy of the document with all sections, see nature.com/documents/nr-reporting-summary-flat.pdf

Behavioural & social sciences study design

All studies must disclose on these points even when the disclosure is negative.

Study description	The study is predominantly quantitative but includes some qualitative components (i.e., participant subjective reports on their vision).
Research sample	Eight cerebrally blind patient participants (females = 2; mean age = 50.25 years; standard deviation [SD] age = 22.76 years; mean education = 17.13 years; SD education = 2.59 years) and eight age and education-matched (confirmed with Mann-Whitney U tests; p > 0.05) healthy control participants (females = 4; mean age = 46.50 years; SD age = 18.69 years; mean education = 16.13 years; SD education = 1.89 years) were recruited. The visual impairment of the patient participants consisted of left homonymous hemianopia (N = 2), right homonymous hemianopia (N = 2), left homonymous inferior quadrantanopia (N = 3), and right homonymous superior quadrantanopia (N = 1).
Sampling strategy	Participant sample size was determined by the availability of cerebrally blind patient participants. Within subject sample size (i.e., the number of trials acquired per participant during the experimental task) was estimated according to pilot testing with patient and healthy participants.
Data collection	All experimental sessions were completed in a windowless, temperature-controlled room at the National Institutes of Health, Bethesda, Maryland, USA. Each study session lasted approximately 2.5 hours, including a health exam, instructions, and task breaks. During the behavioral session, the experimenters were positioned behind the participant to monitor behavior and deliver task instructions. During the MEG session, the experimenters were positioned outside the MEG shielded room and monitored and communicated with the participant via a closed-circuit television (COLOR CCD Camera VCC-3912; Sanyo Electric Co. Ltd.) and intercom console system (VSM MedTech Ltd.). Head-fixed (SR Research Head Support; SR Research, Inc.) monocular pupillometry and eye tracking were acquired with the EyeLink 1000 Plus (sampling rate = 1000 Hz; SR Research, Inc.). MEG data were recorded using a CTF 275 MEG system (sampling rate = 1200 Hz; CTF Systems, Inc., Canada) composed of a whole-head array of 275 radial 1st order gradiometer sensors housed in a magnetically shielded room (Vacuumschmelze GmbH & Co. KG, Germany). A whole brain structural T1-weighted MRI (magnetization prepared – rapid gradient echo [MPRAGE]) was acquired with a 3T MR750 MRI (General Electric, Inc.) and a 32-channel head coil (Nova Medical, Inc.).
Timing	Start collection: 2023/10/17; End collection: 2024/06/20
Data exclusions	Four additional patient participants were recruited but they were not included in analyses due to low data sample size (two participants), poor behavioral performance (one participant made incorrect keypresses and did not maintain central fixation), and not meeting the definition of cerebral blindness (one participant experienced visual impairment due to a chiasmal tumor).
Non-participation	No participant declined to participate.

Randomization	Participants were not allocated to experimental groups.

Reporting for specific materials, systems and methods

We require information from authors about some types of materials, experimental systems and methods used in many studies. Here, indicate whether each material, system or method listed is relevant to your study. If you are not sure if a list item applies to your research, read the appropriate section before selecting a response.

Materials & experimental systems

n/a	Involved in the study
☒	☐ Antibodies
☒	☐ Eukaryotic cell lines
☒	☐ Palaeontology and archaeology
☒	☐ Animals and other organisms
☒	☐ Clinical data
☒	☐ Dual use research of concern
☒	☐ Plants

Methods

n/a	Involved in the study
☒	☐ ChIP-seq
☒	☐ Flow cytometry
☒	☐ MRI-based neuroimaging

Plants

Seed stocks	*Report on the source of all seed stocks or other plant material used. If applicable, state the seed stock centre and catalogue number. If plant specimens were collected from the field, describe the collection location, date and sampling procedures.*
Novel plant genotypes	*Describe the methods by which all novel plant genotypes were produced. This includes those generated by transgenic approaches, gene editing, chemical/radiation-based mutagenesis and hybridization. For transgenic lines, describe the transformation method, the number of independent lines analyzed and the generation upon which experiments were performed. For gene-edited lines, describe the editor used, the endogenous sequence targeted for editing, the targeting guide RNA sequence (if applicable) and how the editor was applied.*
Authentication	*Describe any authentication procedures for each seed stock used or novel genotype generated. Describe any experiments used to assess the effect of a mutation and, where applicable, how potential secondary effects (e.g. second site T-DNA insertions, mosiacism, off-target gene editing) were examined.*
